# CKAP2L, a crucial target of miR-326, promotes prostate cancer progression

**DOI:** 10.1186/s12885-022-09762-3

**Published:** 2022-06-17

**Authors:** Qi Li, Mo Yan, Chunhui Wang, Kaibin Wang, Guochang Bao

**Affiliations:** 1 Tianjin Medical University, Tianjin, China; 2 Departments of Urology, Affiliated Hospital of Chifeng University, Chifeng, China; 3 Urology Research Center, Chifeng University, Chifeng, China

**Keywords:** CKAP2L, Prostate cancer, Cell cycle arrest, miR-326, Migration, Invasion, Proliferation

## Abstract

**Background:**

The overexpression of aberrant cell cycle signaling pathway associated protein has been implicated in multiple malignancies and the identification of all-important one among is the crux of the precise targeted therapy. CKAP2L (Cytoskeleton Associated Protein 2 Like) plays a newish role in cancer progression through activation of the process of cell cycle and mitosis. In this study, we aim to delineate the prominent dysregulated expression of CKAP2L and comprehensively reveal its deregulation in prostate cancer.

**Method:**

CKAP2L expression was examined in the normal and tumor tissues of prostate cancer patients with RT-QPCR and Western blot. IHC showed the different expression in normal prostate tissue, tissue of BPH, low Gleason Score and high Gleason Score prostate cancer patients. Transwell, colony formation, MTT and flow cytometry were performed to detected the changes in cellular function in vitro. The xenograft model was conducted for the changes in vivo. Dual luciferase and RIP proved the binding relation between CKAP2L and miR-326.

**Results:**

In multiple datasets, CKAP2L was found upregulated and positively associated with Gleason grade and poor clinical outcomes of patients. shRNA mediated silence of CKAP2L suppressed cell proliferation, impaired monolayer formation, inhibited cell invasion. CKAP2L was confirmed to be the direct target of miR-326, which had a carcinostatic effect by binding the 3’untranslated regions (3’UTRs) of CKAP2L mRNA. The deletion of CKAP2L resulted in reduced expression of genes involved in the mitotic cell cycle such as multiple cyclin-dependent kinases and cyclins, but also several genes encoding proteins involved in chromosome segregation and spindle assembly.

**Conclusion:**

Taken together, CKAP2L plays a carcinogenic role in prostate cancer by regulates the expression of cycle-associated proteins.

**Supplementary Information:**

The online version contains supplementary material available at 10.1186/s12885-022-09762-3.

## Background

Prostate cancer is a frequently detected cancer in the United States for men over 50 years of age [1]. There were 248,530 new cases of prostate cancer in the United States in 2020. The number reached the historical high and remained the highest number of new cancer cases in male [2]. Existing treatments such as traditional androgen deprivation therapy (ADT) could only delay the progression of prostate cancer at an early stage. Despite initial high response rates, cancer management with ADT is of limited duration, eventually the patients will progress to Castration-resistant prostate cancer (CRPC) [3]. There is an urgent need for a novel treatment to delay the progression of prostate cancer to CRPC.

CKAP2L has been described as Radmis (radical fiber and mitotic spindle protein) according to the location and to the mitotic spindle that is considered to be nonsubstitutable to neural stem or progenitor cell division [4]. The results from the present investigation demonstrated that loss-of-function mutations in CKAP2L is one of the major factors involved in Filippi syndrome. Due to the overexpression of CKAP2L leads to conspicuous increase in cell mitosis, there is a tight association between its expression and appropriate mitosis of neural precursor [5]. To date, however, only a few studies comprehensively investigated the expression and function in cancer progression especially in prostate cancer.

Furthermore, microRNAs (miRNAs) have been reported to abnormally expressed in various types of cancers [6]. Mechanically, they directly binding to the 3′UTR of targeted mRNA to degrade targeted mRNA or inhibit translation [7]. Prior studies have found that the deregulation of miRNAs expression can lead to the changes of oncogenes and tumor suppressor genes, thus affecting cell proliferation, migration, invasion and apoptosis in prostate cancer [8].

In the present study, we elucidated the expression of CKAP2L and its relation to the cell cycle signaling pathway demonstrated the CKAP2L regulation by miRNA in prostate cancer.

## Materials and methods

### Clinical samples and cell culture

There were 5 paracancerous tissues from patients with prostate cancer. Other clinical samples were obtained from 5 patients with BPH (benign prostatic hyperplasia), 12 prostate cancer patients with low Gleason score and 12 patients with high Gleason score came from the Second Hospital of Tianjin Medical University. All patients provided informed consent for the study, which got the approval of Research Ethics Committee of the Second Hospital of Tianjin Medical University. The human prostate cancer cell lines, LNCaP, 22Rv1, C4-2, DU145, PC-3, and human benign prostatic hyperplasia cell line BPH-1, were obtained from the American Type Culture Collection (ATCC, Manassas, VA, USA). 22Rv1 and DU145 cells were maintained in RPMI-1640 (Gibco, Waltham, MA, USA) supplemented with 10% fetal bovine serum (FBS) and penicillin/streptomycin at 37 °C, 5% CO_2_. LNCaP-AI cells were established by LNCaP cells in RPMI medium supplemented with 10% charcoal–dextran-stripped FBS (CD-FBS) for more than 12 months at 37 °C, 5% CO_2._

### Protein extraction and western bolt assays

Western blot was performed with whole-cell protein lysates in RIPA lysis buffer (Thermo Fisher Scientific, Inc.) and lysed by centrifugation at 14,000 rpm for 30 min at 4 °C. The polyacrylamide gel was prepared by Solarbio kit (P1200, Solarbio, China) Equal amounts of 30 µg proteins were fractionated by SDS–PAGE, then transfected to polyvinylidene difluoride membranes. After blocked in 5% Skimmed milk for 1 h, the membranes were incubated with primary antibodies overnight at 4 °C. Then secondary antibodies were incubated for 1 h in room temperature. Use immobilon western chemilum hrp substrate (WBKLS0100, Millipore) to exposure and photography. All experiments were performed three repetitions.

### Stable and transient transfection

For shRNA transfection, PC-3 and LNCaP-AI cells were transfected with lentiveiral shRNA purchased from GENECHEM (Shanghai, China) when the cells reaching 40–60% confluency. Plasmids constructed by GENECHEM (Shanghai, China) were transiently transfected into prostate cancer cells using Lipofectamine2000 (Thermo Fisher Scientific, Inc.) For preparation, cells were grown in complete medium for at least 24 h, and washed 3 times by phosphate buffered saline (PBS; pH 7.4) before transfection. Stable expression was selected with puromycin after transfection. All experiments were performed three repetitions.

### RNA extraction and Quantitative Real Time Polymerase Chain (QRT-PCR) assay

To detect the expression levels of miR-326 and CKAP2L, qRT-PCR assay was performed. Total RNA was extracted from the cells with TRIzol (Invitrogen Life Technologies, USA) according to the manufacturer’s instructions. The reverse transcription kit used in the reverse transcription process was purchased from Thermo Fisher Scientific. Then cDNA amplification was conducted using the SYBR-Green PCR Master Mix (Roche) with Applied Biosystems 7900 Real Time PCR System (Thermo Scientific). The relative expression level was analyzed using 2^−ΔΔCt^ method and normalized with U6 and GAPDH as endogenous control. The primers used in this study was listed in Table 1. All experiments were performed three repetitions.

**Table 1:** All primer sequences used in this study

Target gene	Forward (5’-3’)	Reverse (5’-3’)
miR-326	GCCGAGCCTCTGGGCCCTTC	CAGTGCGTGTCGTGGAGT
CKAP2L	GAGCCAAAACACCAAGCCTTA	GGAGTTTAATGCTGATGGACCTT
GAPDH	CGGAGTCAACGGATTTGGTCGT	GGGAAGGATCTGTCTCTGACC
U6	CTCGCTTCGGCAGCACA	AACGCTTCACGAATTTGCGT

### Immunohistochemistry staining

CKAP2L IHC was performed with the standard streptavidin–biotin-peroxidase complex method by the immunohistochemistry kit (pv-6000, ZSGB-BIO, China). The nuclear accumulation of CKAP2L was assessed according to the ratio of prostate cancer cells with positive nuclear staining. The results were scored by the positive cell rate and staining intensity.

### Cell proliferation assay

Transfected cells were digested, resuspended and then plated into 96-well plates at a density of 8 × 10^3^ cells/well with six repeats. At 0, 24, 48, and 96 h, 10 μL of MTT regent (5 mg/mL) was added into per well and cells were incubated at 37 °C for 2 h. Thereafter, the supernatant was removed and the precipitate was solubilized in 200 μL of dimethyl sulfoxide (DMSO). Finally, the absorbance value of each well was measured at a wavelength of 490 nm.

### Colony formation assay

The colony formation assays were performed to test the colony formation ability of cells. 500–1000 cells were seeded per well in 60 mm plates and grown for two weeks. Then cells were fixed with methanol for 15 min and stained with 1% crystal violet-acetic acid solution for 20 min, colonies were visualized and quantitated by ImageJ. All experiments were performed three repetitions.

### Transwell assay

Migration assay: Cells on logarithmic growth phase were performed with serum starvation for 24 h. On the next day, cell suspension with concentration of 4 × 10^4^ cells/mL was prepared. 0.2 mL of suspension was added into the Transwell inserts, and 800 μL of pre-cooled 1640 containing 10% FBS/CD-FBS was placed out of the inserts. After 48 h of incubation in 5% CO_2_ at 37 °C, unmigrated cells were wiped off with a cotton swab, meanwhile, cells migrated out of the inserts were fixed by methanol at 4 °C for 30 min and stained in 0.1% crystal violet for 20 min. Images were captured with an inverted microscope, and three fields were randomly selected for cell count. All experiments were performed three repetitions.

Invasion assay: Roughly 2 × 10^4^ cells were transfused into the upper chambers with Matrigel matrix (Corning, NY, USA) precoated. 800 μL of pre-cooled 1640 containing 10% FBS/CD-FBS was placed in the lower chambers. Following steps were similar with migration assay as mentioned above. All experiments were performed three repetitions.

### RNA-Binding Protein Immunoprecipitation Assay (RIP)

RIP assay was performed using the EZ-Magna RIP™ RNA-Binding Protein Immunoprecipitation Kit (Millipore 3,480,215, Billerica, USA) as described by the manufacturer protocol. Briefly, cells were lysed with complete RIP lysis buffer. 100 μL whole cell lysate from each group were incubated with RIP buffer containing magnetic beads conjugated to mouse anti-Ago2 antibody (ab186733, 1:50, Abcam, United Kingdom), or negative control normal rabbit IgG (Millipore, USA). Consequently, the immunoprecipitation was digested by incubating with proteinase K to purify the immunoprecipitated RNA. Purified RNA was reverse transcribed into cDNA and subjected to qPCR to examine the presence of CKAP2L mRNA. All experiments were performed three repetitions.

### Primers and antibodies

All primer details are listed in Table 1. The information of antibody in western blot and IHC are listed in Table 2.

**Table 2:** Antibodies and concentrations used in Western Blot and IHC in this study

Antibodies	Source, Identifier	Western blot	IHC
CKAP2L	Thermo Fisher Scientific, PA5-58,778	1:1000	1:200
MAD2L1	Abcam, ab10691	1:500	/
PLK1	Abcam, ab189139	1:1000	/
AURKB	Abcam, ab45145	1:5000	/
BIRC5	Abcam, ab76424	1:5000	/
CCND1	Abcam, ab16663	1:200	/
SMC4	Cell Signaling Technology, 5547S	1:1000	/
KIF2C	Abcam, ab187652	1:1000	/
GAPDH	Abcam, ab181602	1:10,000	/
Cleaved Caspase-3	Abcam, ab2302	/	1:10
Ki-67	Abcam, ab16667	/	1:200

### Luciferase reporter assay

The putative has-miR-326 binding sites in 3′UTR of CKAP2L, as well as the mutant binding sites were sub-cloned into pmirGLO luciferase vector (Promega, Madison, WI, USA) to construct luciferase expression plasmids WT-CKAP2L and MUT-CKAP2L. Then co-transfected them into prostate cancer cells with miR-326 mimic or miR-326 mimic NC. Lipofectamine 2000 was used for transfection according to the instructions. After 48 h, a luminometer (Beckman Coulter LD400, CA, USA) was used to detect the luciferase activity. All experiments were performed three repetitions.

### Cell cycle assessment

PC3 and LNCaP-AI cells were harvested, fixed with 70% ethanol, briefly washed with PBS/1% FBS, and then incubated with 10 mg/mL RNAse A and 50 mg/mL propidium iodide in PBS plus 1% Tween 20 for 30 min at 37 °C in the dark. Flow cytometric analysis was performed using FACS Calibur flow cytometer (BD Biosciences, Franklin Lakes, NJ, USA). The cell fractions in the respective cell cycle phases were calculated using Cell Quest Pro software (BD Biosciences, USA). All experiments were performed three repetitions.

### Animal model

To check the inhibition effect on tumor growth after CKAP2L depletion, CKAP2L was downregulated by shRNA-mediated knockdown in PC3 cells. Then the shCKAP2L transfected cells and their counterparts were subcutaneously injected into the Balb/c nude mice (6- to 8-week-old). The tumor weights were measured after the tumor formation.

### Statistical analysis

The datasets were obtained from databases. The MSKCC dataset was downloaded from Cancer Genomics at cBio—Prostate Cancer (MSKCC)—Genomic Data. The TCGA dataset was downloaded from UCSC Xena (xenabrowser.net). Associations between CKAP2L or miR-326 expression and clinicopathological parameters were achieved by nonparametric analysis. Kaplan–Meier curves were fitted to disease free survival data by Hiplot | Emerging biomedical data visualization toolkit. The samples were divided in to two groups by the mean of the CKAP2L expression. Statistical difference of two groups with uneven population was calculated by Mann–Whitney test (not subject to normal distribution) or unpaired t-test (subject to normal distribution). For the other comparison of two groups such as qPCR, numbers of transwell or colony formation, we performed student's t test. The correlation of expression was analyzed by Spearman correlation test. One-way ANOVA was used for multiple groups comparison, and the error bars mean ± SD. Differences were considered significant when *p* < 0.05. Analyses were performed using GraphPad Prism 8 software (Intuitive Software for Science) and *R*-3.6.0.

## Result

### CKAP2L is upregulated in pan-cancer and associated with high Gleason score and poor survival in prostate cancer

**Fig. 1:**
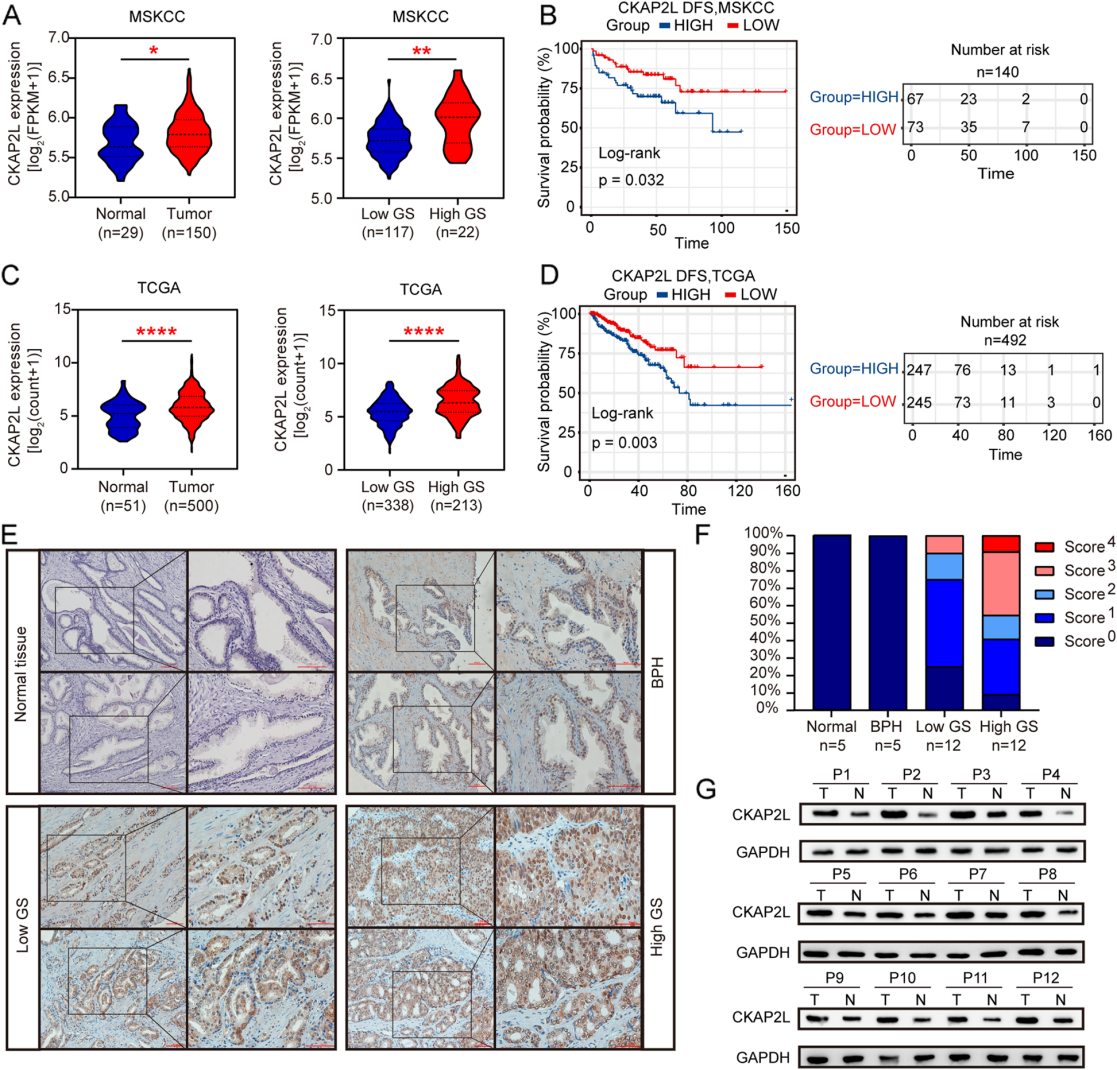
mRNA expression of CKAP2L in TCGA and MSKCC prostate cancer cohort with GS. (**A, C**) CKAP2L is overexpressed in prostate cancer and correlated with high Gleason Score. (**B, D**) Higher CKAP2L mRNA expression was significantly associated with poorer prognosis in MSKCC and TCGA cohort as Kaplan–Meier curves showing. (**E, F**) The immunohistochemistry of CKAP2L in BPH, low Gleason Score (GS < 7) and high Gleason Score (GS > 7) prostate cancer samples. (**G**) CKAP2L protein expression in prostate tumor (T) and adjacent normal tissue(N) samples from 12 high Gleason Score (GS > 7) prostate cancer patients. *p < 0.05, **p < 0.01, ***p < 0.001, ****p < 0.0001, ns: *p* > 0.05

CKAP2L mRNA expression levels were analyzed in TCGA with Timer to investigate CKAP2L expression over a cancer wide range. As shown in Figure S1A, the CKAP2L expression patterns in tumor and adjacent normal were significantly different. In both TCGA cohort and MSKCC, CKAP2L was also concordantly elevated in prostate cancer tumor samples compared with adjacent normal prostate tissue The expression level of CKAP2L increases with the malignancy of the disease judged by Gleason score (Fig. 1A and C). Meanwhile, survival analysis in both TCGA and MSKCC revealed that high CKAP2L expression associated tightly with poor clinical outcomes (Fig. 1B and D).

This indicated that CKAP2L might play a pivotal role in prostate cancer progression. Therefore, 5 paracancerous tissues from patients with prostate cancer, 5 BPH tissues, 12 prostate cancer tissues form patients with high and low Gleason score, respectively, were selected and detected by Immunohistochemistry (Fig. 1E, F). Consistent with the results of immunohistochemistry, western blot of samples from prostate cancer patients showed that CKAP2L expression remarkably upregulated in protein-level with the prostate cancer progression (Fig. 1G).

### CKAP2L knockdown exerts anti-oncogenic effect in vitro and in vivo

**Fig. 2:**
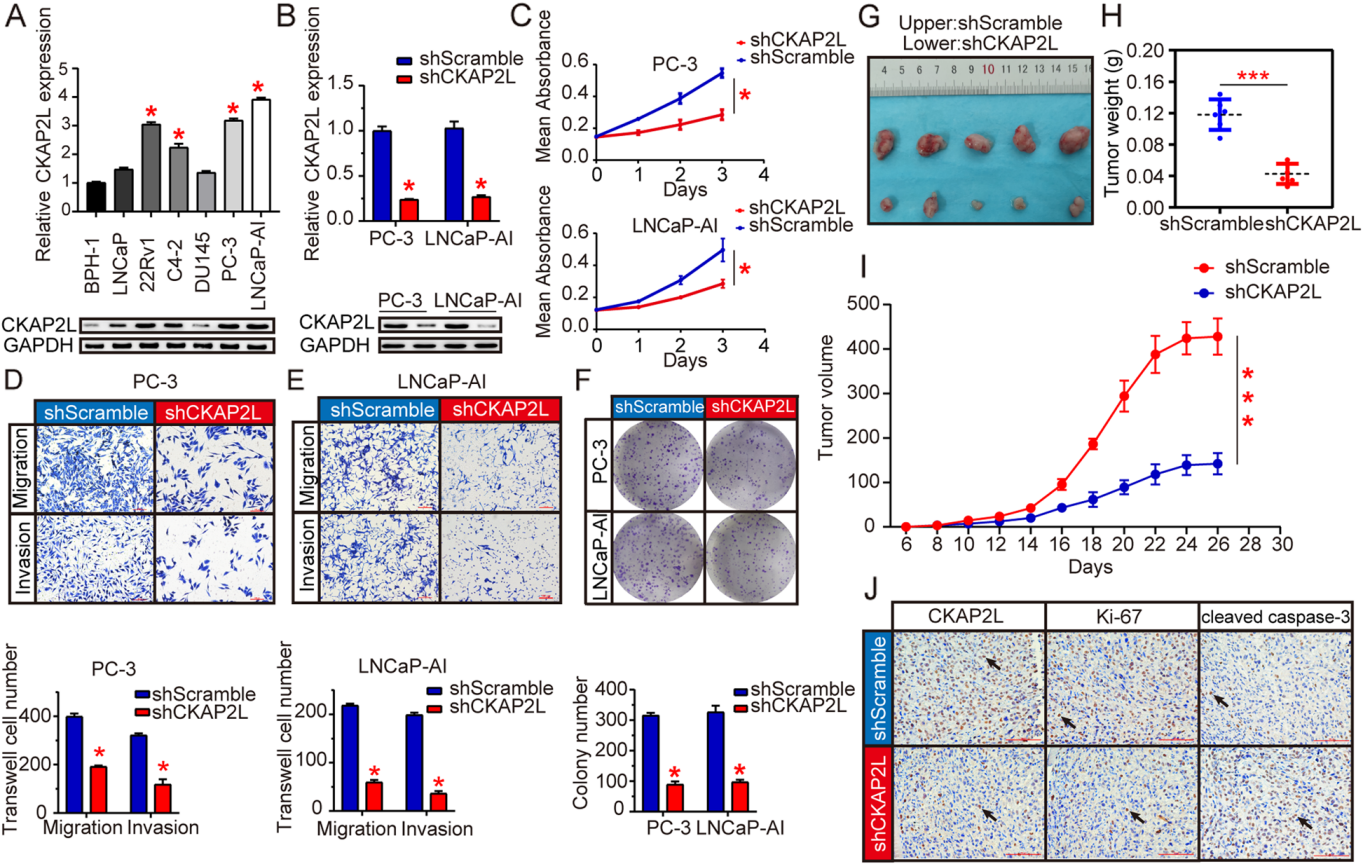
CKAP2L plays an oncogenic role in vitro and vivo. (**A**) CKAP2L mRNA and protein expression in BPH-1, 22Rv1, C4-2, PC-3, DU145 and LNCaP-AI cell lines. (**B**) CKAP2L expression in mRNA and protein level after shCKAP2L transfected in PC-3 and LNCaP-AI cells. (**C**) Silencing CKAP2L suppressed PC-3 and LNCaP-AI cells proliferation. (**D, E**) CKAP2L deletion inhibited cell migration and invasion ability in PC-3 and LNCaP-AI cells. (**F**) CKAP2L knockdown decreased monolayer colony formation ability of the prostate cancer cells. (**G-I**) Knockdown of CKAP2L by shRNA remarkably suppressed the xenograft formation according to the tumors weight and volume in nude mice compared with negative control. (**J**) Immunohistochemistry showed that compared with the control group, knocking down CKAP2L significantly decreased Ki-67 and in nude mouse tumors, while the expression of cleaved caspase-3 increased. *p < 0.05, ***p < 0.001

Chiefly, the expression of CKAP2L in cell lines was verified by RT-qPCR and western blot which showed that compared with BPH-1, CKAP2L in 22Rv1, C4-2, PC-3, and LNCaP-AI cells upregulated notably in mRNA and protein levels while CKAP2L in LNCaP and DU145 not (Fig. 2A). To further investigate the biological function of CKAP2L in human prostate cancer cell lines, we performed sh-RNA mediated CKAP2L stable knockdown in the highest expression of CKAP2L prostate cancer cell line (Fig. 2B). CKAP2L depletion by shRNA in PC-3 and LNCaP-AI significantly reduced cell proliferation (Fig. 2C). Cell migration and invasion ability was also impaired after CKAP2L knockdown (Fig. 2D and E) Constantly, CKAP2L silencing suppressed monolayer colony formation ability of these cells (Fig. 2F).

To further investigate the impact of CKAP2L depletion in vivo, shCKAP2L transfectants performed prior were inoculated subcutaneously into the nude mice. After 26 days, analysis of the results showed that the tumor volume was remarkably reduced in the shCKAP2L group compared with shScramle group (Fig. 2G). Furthermore, the significant difference of the tumor weights between treated group and negative control group corresponded to the volume results as well (Fig. 2H). Besides, the tumor growth rates were also detected (Fig. 2I). The expression of CKAP2L and proliferation marker Ki-67, were detected to verify the ability of tumor proliferation. Meanwell, the depletion of CKAP2L caused apoptotic cell death assessed by cleaved caspase-3 expression. Results indicated that CKAP2L silencing down regulated the expression of Ki-67 and cleaved caspase-3, which implied the reducing ability of tumor proliferation and significance increase in apoptotic cell death as well (Fig. 2J).

### miR-326 targets CKAP2L and represses its expression

**Fig. 3:**
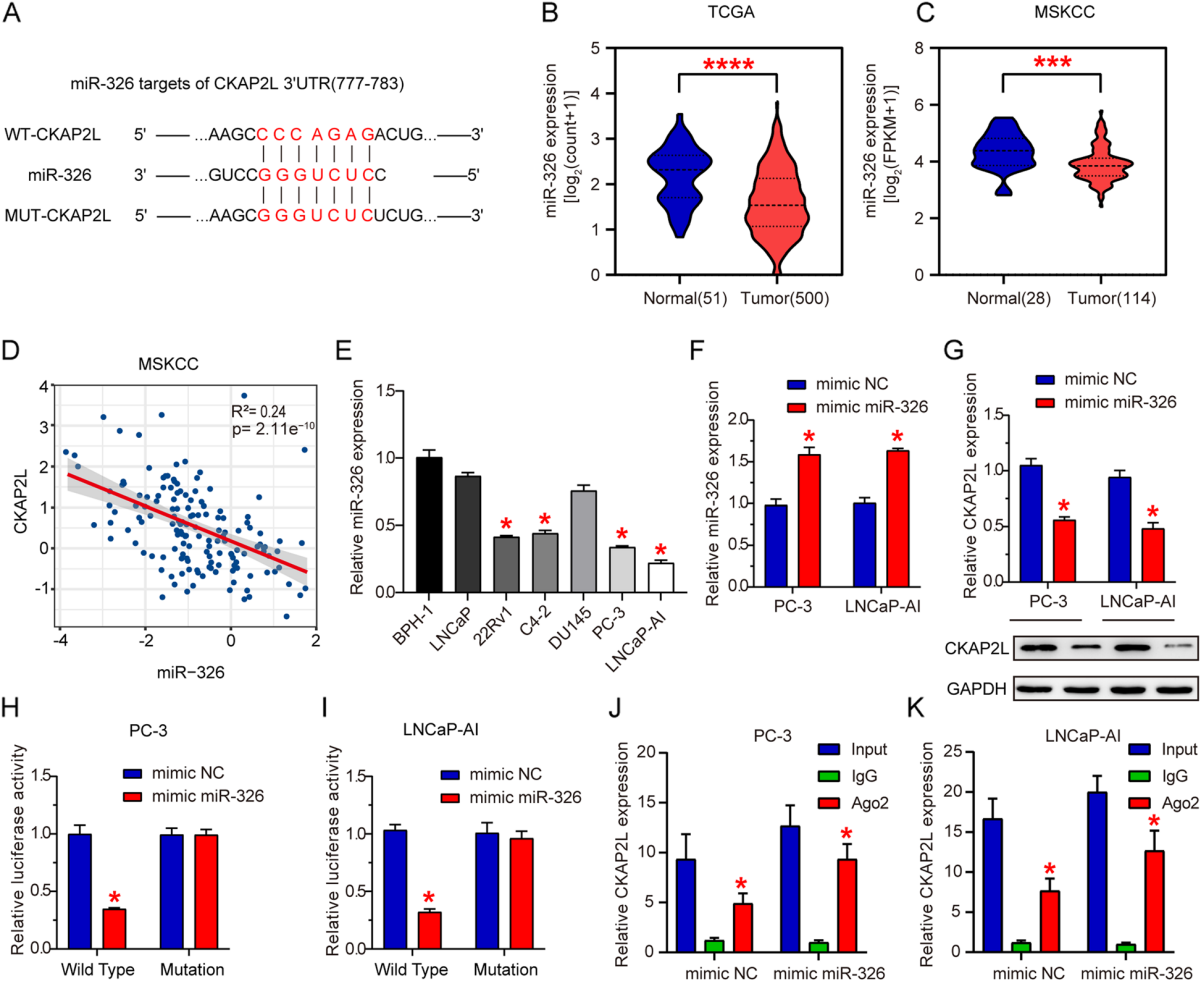
miR-326 targets CKAP2L and inhibits its expression. (**A**) The binding sites of miR-326 targeting CKAP2L were predicted. (**B, C**) In both TCGA and MSKCC cohorts, expression level of miR-326 in tumor and normal tissues (**D**) CKAP2L mRNA is negatively associated with miR-326. (**E**) Expression of miR-326 in BPH-1, 22Rv1, C4-2, PC-3, DU145 and LNCaP-AI cell lines. (**F**) miR-326 expression after mimic miR-326 transfected in PC-3 and LNCaP-AI cells. (**G**) CKAP2L mRNA and protein expression in mimic miR-326 transfected PC-3 and LNCaP-AI cells. (**H, I**) Dual-luciferase assay was performed to verify the binding relationship between CKAP2L mRNA and miR-326. (**J, K**) RIP was conducted to demonstrate that miR-326 directly interacts with CKAP2L mRNA binding sites. The comparison was between the igG and Ago2. *p < 0.05, **p < 0.01, ***p < 0.001, ****p < 0.0001

As predicted in TargetScan, CKAP2L was a downstream target of miR-326 (Fig. 3A). The expression of miR-326 was much higher in normal tissues compared to tumor tissues in both TCGA and MSKCC cohorts (Fig. 3B and C). Besides, the correlation analysis in MSKCC was carried out, which showed a negative association between CKAP2L and miR-326 (Fig. 3D). To verify the expression of miR-326 in prostate cell line, RT-qPCR was performed. The result suggested decreased expression of miR-326 in 22Rv1, C4-2, PC-3 and LNCaP-AI (Fig. 3E). miR-326 expression was significantly increased in the two cells lines after transfection with miR-326 mimic (Fig. 3F). Ectopic expression of miR-326 decreased the mRNA and protein expression of CKAP2L (Fig. 3G). To test whether miR-326 directly binds to the 3’UTR of CKAP2L, dual-luciferase activity assays were performed. Overexpression of miR-326 inhibited the luciferase activities of the reporters containing the wild-type binding sites. On the contrary, the inhibitory effect vanished in the constructs containing mutant binding sites (Fig. 3H and I). Meanwhile, findings concluded by RIP suggested the remarkably up-regulated CKAP2L mRNA expression in miR-326 mimic transfected cells (Fig. 3J and K). All above results suggested that miR-326 targets CKAP2L, leading to its decreased expression.

### miR-326/CKAP2L axis exerts carcinostatic effect in vitro and participated in chromosome segregation and spindle assembly to induce cell cycle G2/M arrest

**Fig. 4:**
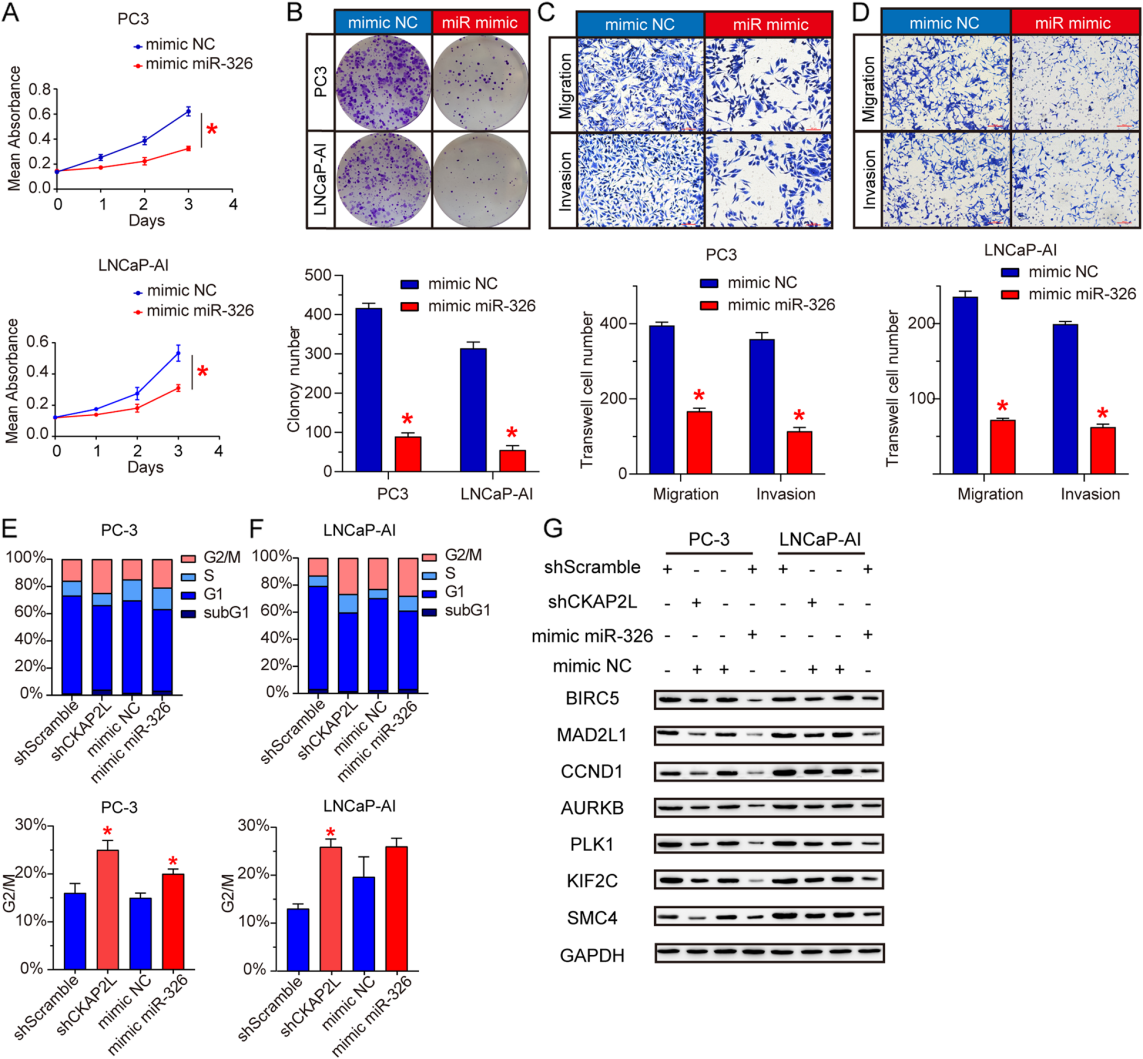
miR-326 plays a carcinostatic role and involves in cell cycle by regulating mitosis. (**A**) mimic miR-326 transfection impaired the cell proliferation in PC-3 and LNCaP-AI cells. (**B-D**) miR-326 overexpression decreased the ability of cell proliferation, monolayer colony formation, migration and invasion in PC-3 and LNCaP-AI cells. (**E, F**) Flow cytometry was carried out to investigate the CKAP2L knockdown or miR-326 overexpression in PC-3 and LNCaP-AI cells. (**G**) Protein levels of several gene involved in chromosome segregation, spindle assembly and cell cycle after CKAP2L knockdown, miR-326 overexpression, or transfected together. *p < 0.05

**Fig. 5:**
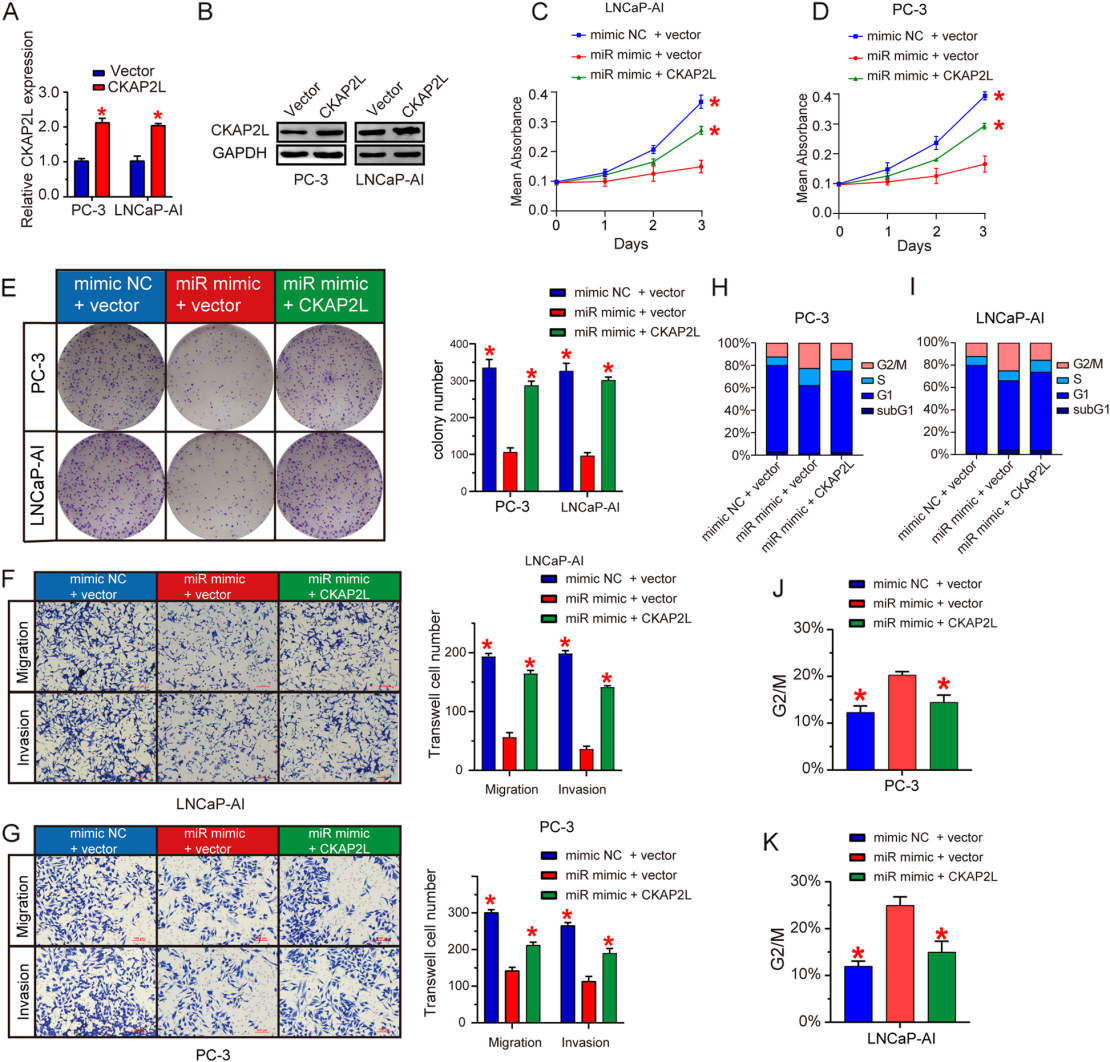
CKAP2L rescue in cells with miR-326 mimic partly restored the cell function. (A) The efficiency of CKAP2L overexpression in mRNA level. (B) The efficiency of CKAP2L overexpression in protein level. (C-G) CKAP2L rescue partly restored the ability of cell proliferation, invasion and migration. (H–K) Flow cytometry was performed to determine the effect of CKAP2L rescue. The results were from group miR mimic + vector comparing mimic NC + vector and miR mimic + CKAP2L. *p < 0.05

The functional role of miR-326 was investigated in PC-3 and LNCaP-AI cells. Ectopic expression of miR-326 significantly suppressed cell proliferation (Fig. 4A) and inhibited the monolayer colony formation ability (Fig. 4B). Overexpression of miR-326 also quench cell invasion and migration ability (Fig. 4C and D). To investigate the impact of CKAP2L depletion on mitotic progression, we detected the cell cycle phase distribution in shScramle and shCKAP2L PC-3 and LNCaP-AI cells. Analysis of the results showed that CKAP2L knockdown remarkably increased the number of G2/M phase cells (Fig. 4E and F). After that, we detected the changes of several genes encoding proteins involved in chromosome segregation, spindle assembly and cell cycle after decreasing CKAP2L expression (Fig. 4G). Furthermore, we performed the rescue experiment to verify the results above-mentioned. CKAP2L overexpression in mir-326 mimic treated cells partly recue the cell function (Fig. 5). The results suggested that CKAP2L depletion significantly affected the ability of cell division by involving mitosis progression.

## Discussion

Overall, our study established the first evidence that CKAP2L was negatively regulated by miR-326. Expanded the function of CKAP2L in prostate cancer by activation of the process of cell cycle and mitosis. Firstly, we verified the high expression of CKAP2L in prostate cancer tissues and cell lines. Then we demonstrated the remarkable decreased ability of cell invasion, migration and proliferation after CKAP2L depletion. In vivo, silencing CKAP2L significantly inhibited the tumor growth rates and final weights. The proliferation and apoptotic marker expression detected in the xenografts verified the results above as well.

CKAP2L is accepted to promote cancer progression by involving in chromosomal instability. In addition, depletion of CKAP2L increased the sensitivity of non-small cell lung cancer (NSCLC) cells to alvocidib, a pan CDK inhibitor, leading to a significant reduction of cell proliferation and an increase in cell death [9]. In glioblastoma cell line, a recent study confirmed that CKAP2L knockdown with siCKAP2L inhibited glioma cell proliferation, migration, invasion, and epithelial-mesenchymal transition [10]. As a crucial transcriptional regulator, the relationship between CKAP2L and cyclin, as well as its downstream related pathways, remains to be solved in the following studies.

Besides, it is instructive that breast cancer, also a hormone-dependent cancer for which various cyclin inhibitors have been successfully approved. A previous study indicated that the next-generation sequencing conduce to reveal the alterations in genes from cyclin pathway in advanced prostate cancer [11]. Preliminary data in prostate cancer suggest that the cyclin pathway plays an important role in the evolution to a castrate-resistant state [12]. Meanwhile, clinical trials with cyclin inhibitors are ongoing in prostate cancer [13]. Hence, according to the studies above and our results, CKAP2L might be a potential therapeutic target or another upstream regulator to mediate cyclin to control the cancer progression.

Furthermore, miR-326 was predicted to directly target CKAP2L mRNA by TargetScan and demonstrated by RIP and dual-luciferase reporter assay in the present study. Overexpression of miR-326 impaired the function of prostate cancer cell proliferation, migration and invasion ability. Flow cytometry showed the accumulation of CKAP2L depletion cells and miR-326 mimic transfected cells in G2/M phase. Moreover, the following western blot results showed that the expression of protein involved in chromosome segregation and spindle assembly were downregulated after CKAP2L knockdown or mimic miR-326 transfected. Therefore, the same effect as knockdown CKAP2L might be achieved by expressing miR-326.

MiRNAs can serve as oncomiRs by targeting tumor suppressor mRNAs that encode oncoproteins. In prostate cancer, previous study shows that miR-34a is a key negative regulator of CD44^+^ prostate cancer cells and establishes a strong rationale for developing miR-34a as a novel therapeutic agent against prostate CSCs [14]. Another study highlighted that USP2a enhances MYC levels through the modulation of specific subsets of microRNAs, suggesting alternative therapeutic strategies for targeting MYC [15]. Numerous preclinical studies utilizing various disease models have tested the use of these new-generation therapeutics, and several miRNA-based therapeutics have advanced into clinical testing [16].

Due to the simple structure and in-depth research in miR, it can be combined with a variety of innovative delivery materials to target the downstream and control the cancer progression. An early study demonstrated the successful inhibition of miR-10b using ASOs in a model of breast cancer. This antimiR resulted in inhibition of metastasis due to rescue of the expression of the anti-metastatic gene HOXD10 [17]. In another recent publication, a cholesterol-modified form of antimiR-221, delivered intravenously to HCC xenografts, showed significant activity in downregulating miR-221 and increasing the levels of its mRNA targets [18]. Meanwhile, using an antimiR against miR-630 and the DOPC delivery platform in an orthotopic model of ovarian cancer, there was a significant reduction in tumor growth and metastasis [19].

Combining to the novel treatments above, our results suggest a possibility of controlling the prostate cancer progression by overexpressing miR-326. As a classic tumor suppressor, miR-326 has been intensively researched in various cancers. A recent study showed miR-326 targeted mitogen-activated protein kinase (MAPK) 1 and colony stimulating factor (CSF)-1 to regulate the cancer cell proliferation and tumor-associated macrophage infiltration in hepatocellular carcinoma (HCC) [20]. In another study, enforced expression of miR-326 attenuated the promotive effect of PCAT1 on oesophageal squamous cell carcinoma (ESCC) cell growth [21]. There was a similar study reported in lung cancer that demonstrated that miR‐326/Sp1/KLF3 regulatory axis is involved in the development of lung cancer [22]. Therefore, targeting CKAP2L by delivering miR-326 might be a feasible treatment to control prostate cancer progression.

## Conclusions

In summary, our study revealed that overexpression of miR-326 can inhibit cell proliferation, migration and invasion in prostate cancer cell line by regulating CKAP2L expression, which can be further investigated as a potential treatment target and a novel diagnosis marker.

## Supplementary Information

**Additional file 1:** (PDF 18973 kb)

**Additional file 2:** (PDF 235 kb)

## Data Availability

The RNA sequencing results and clinical information were obtained from GSE21034 GEO dataset (https://www.ncbi.nlm.nih.gov/geo/query/acc.cgi?acc=GSE21034) and TCGA database (https://xenabrowser.net/datapages/).
