# Supplementary figures and images for: CKAP2L, a crucial target of miR-326, promotes prostate cancer progression
Source: BMC Cancer. 2022 Jun 17;22:666. doi: 10.1186/s12885-022-09762-3 (PMC9206381; doi:10.1186/s12885-022-09762-3)

A

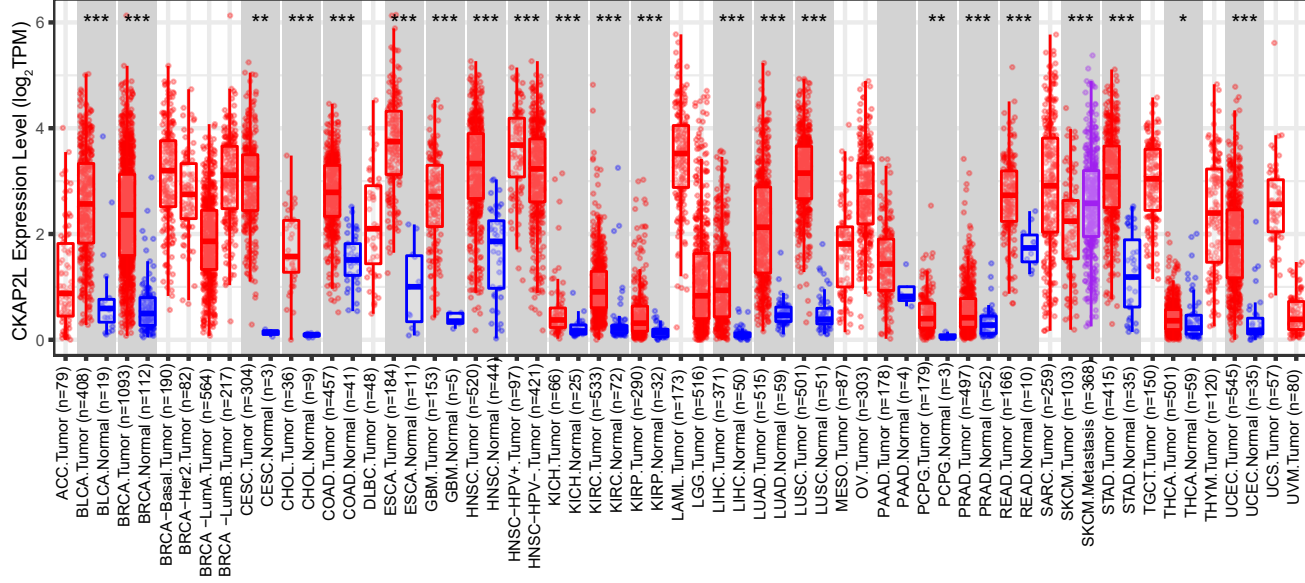

Supplement: Supplementary file 1 — Additional file 1: (PDF 18973 kb) [file 12885_2022_9762_MOESM1_ESM.pdf]
