# Supplementary material for: CKAP2L, a crucial target of miR-326, promotes prostate cancer progression
Source: BMC Cancer. 2022 Jun 17;22:666. doi: 10.1186/s12885-022-09762-3 (PMC9206381; doi:10.1186/s12885-022-09762-3)

Figure_1F
P1-4
CKAP2L

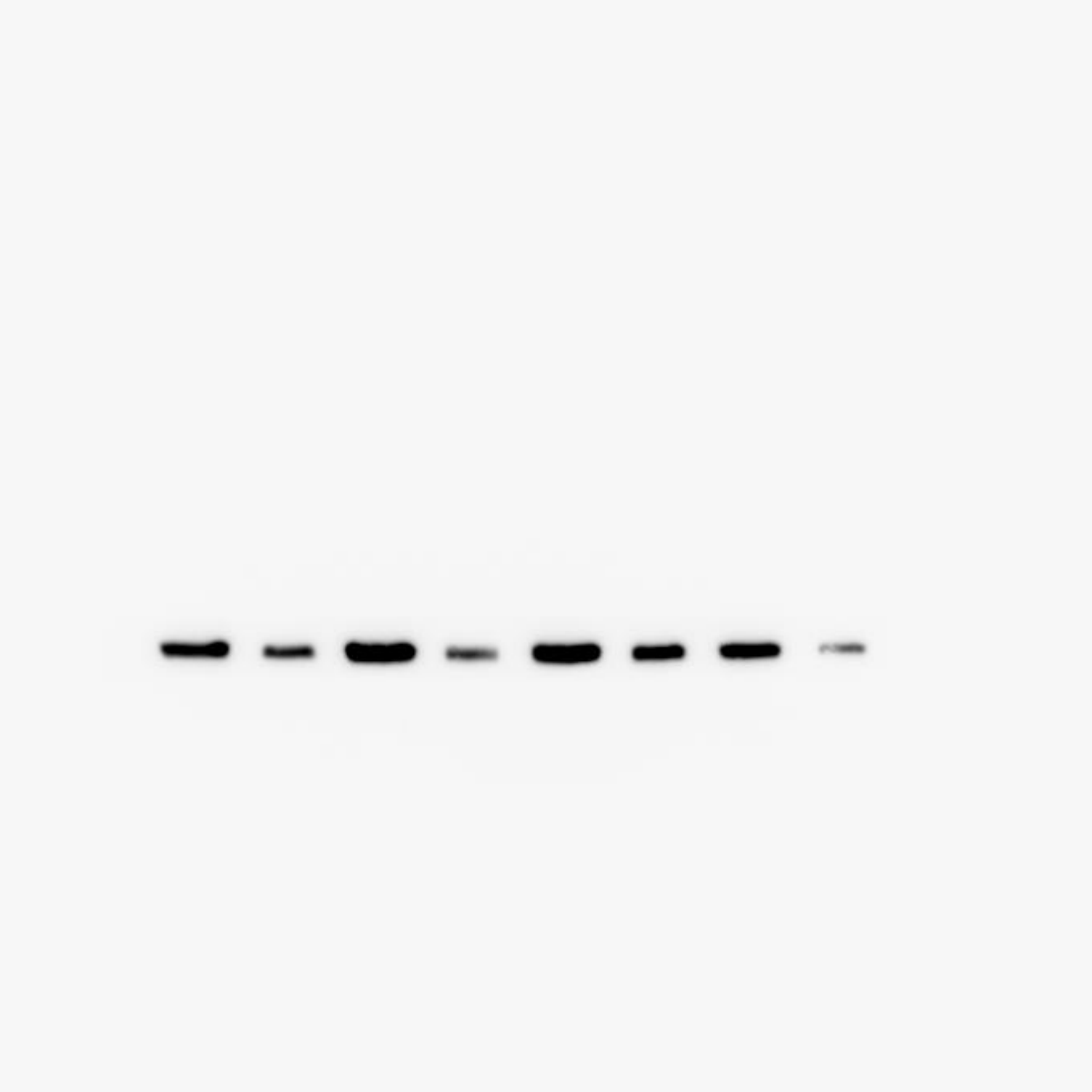

Figure_1F
P1-4
GAPDH

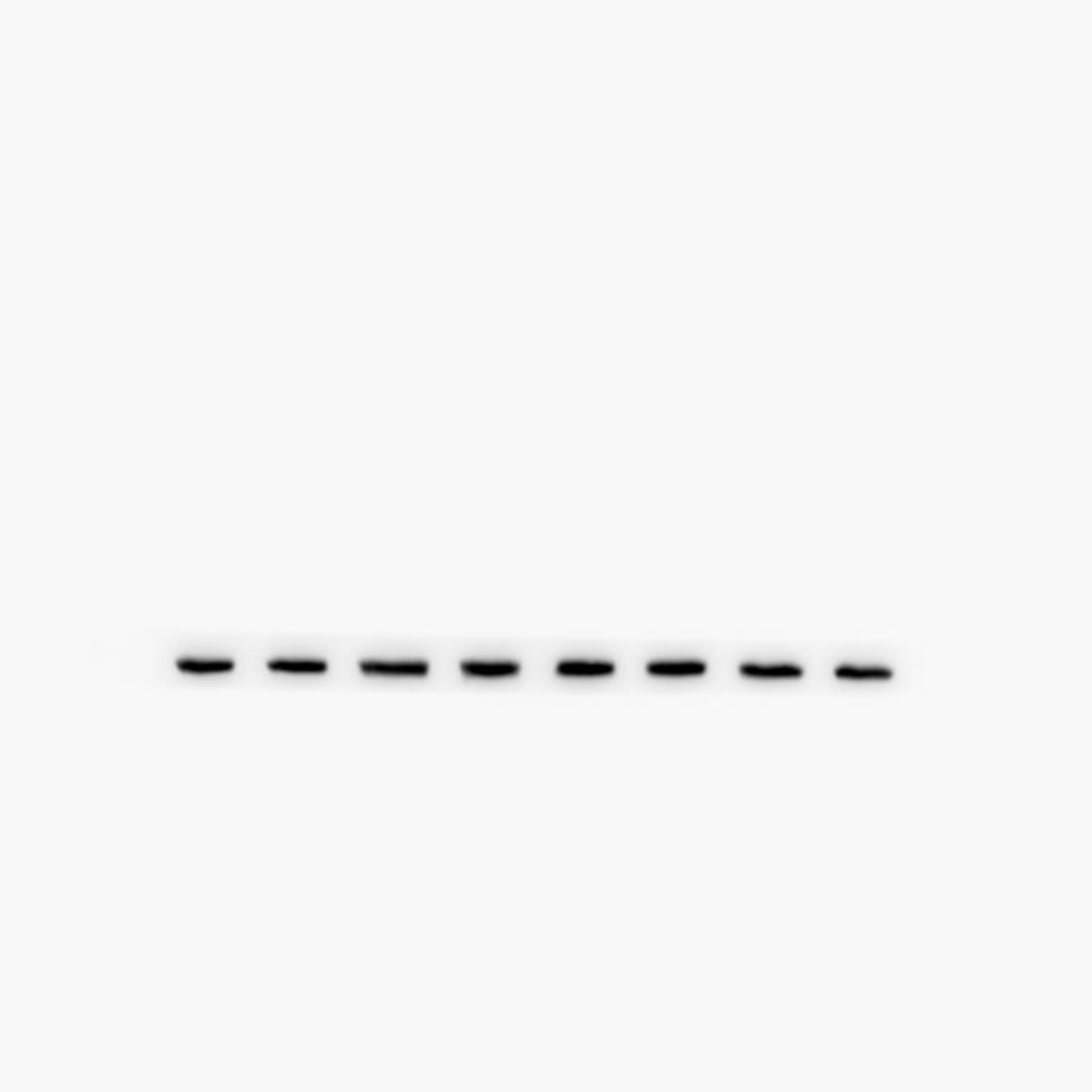

Figure_1F
P5-8
CKAP2L

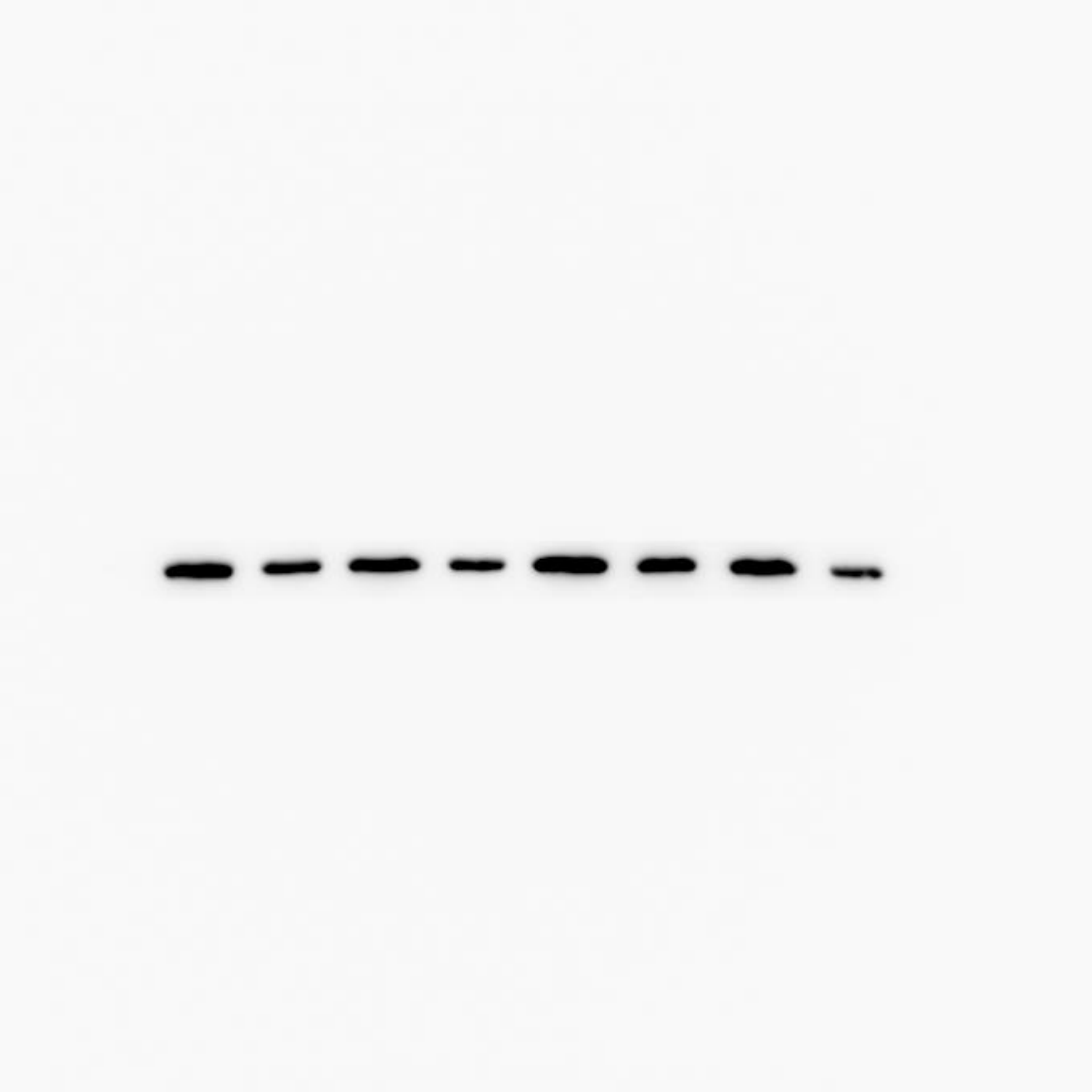

Figure_1F
P5-8
GAPDH

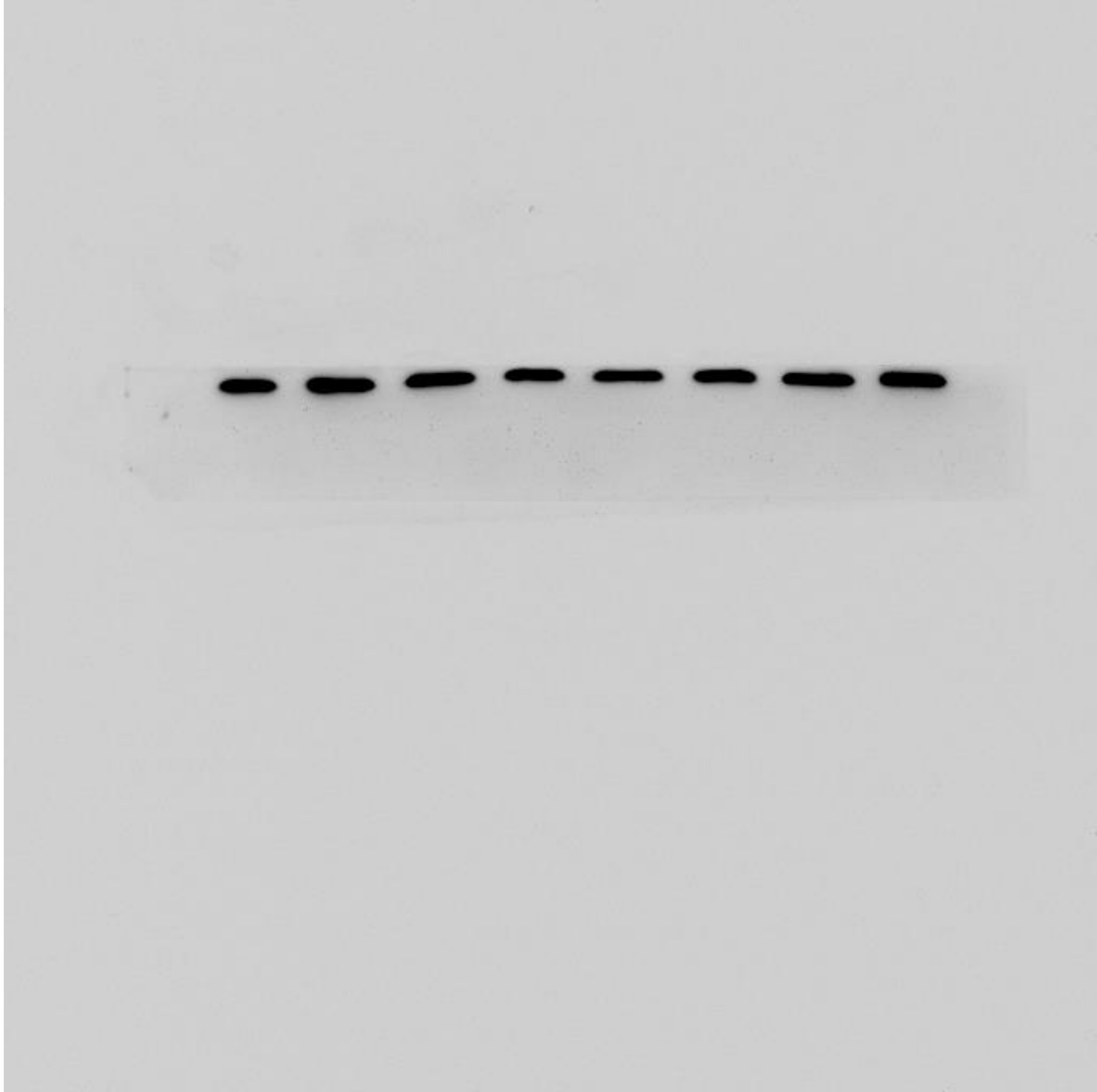

Figure_1F
P9-12
CKAP2L

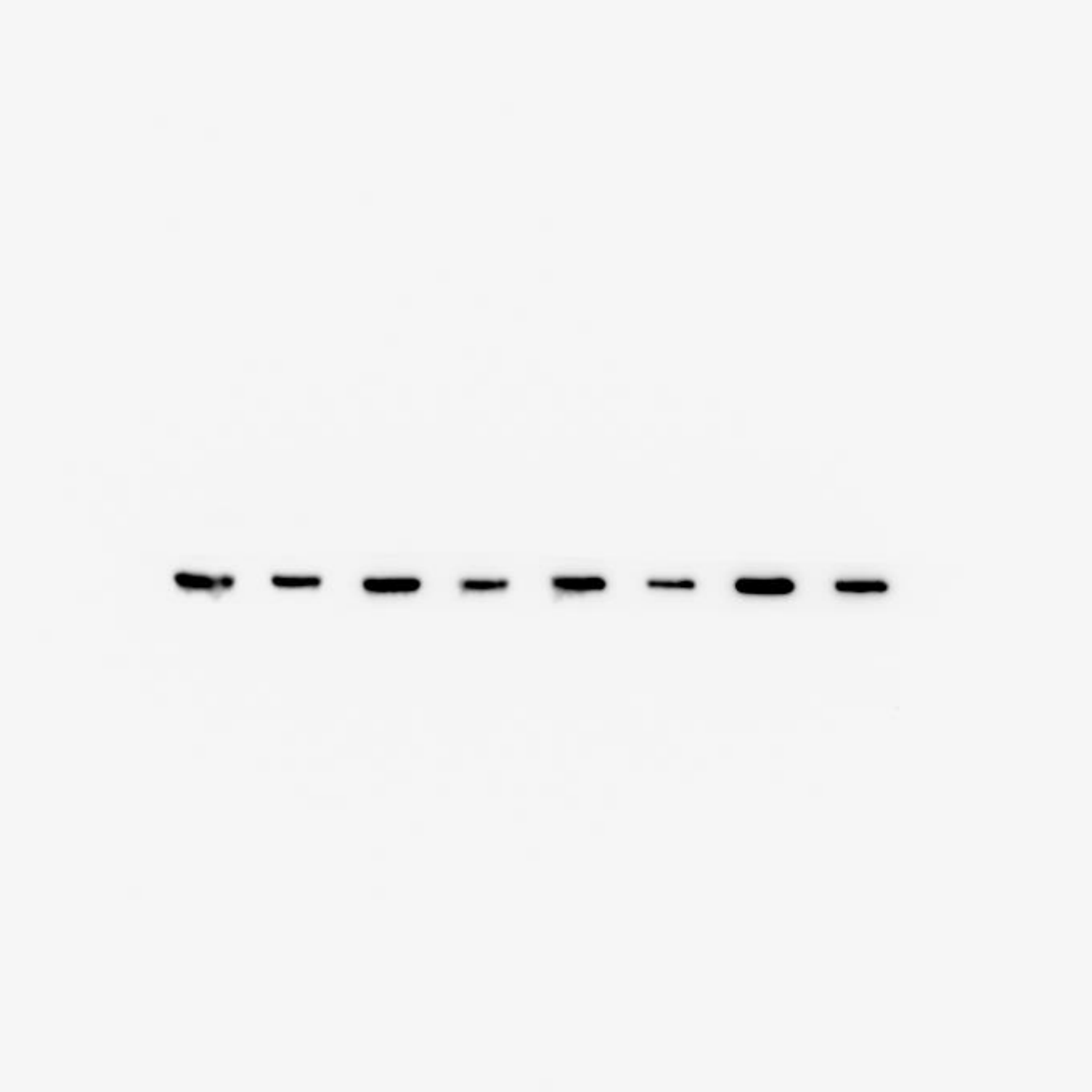

Figure_1F
P9-12
GAPDH

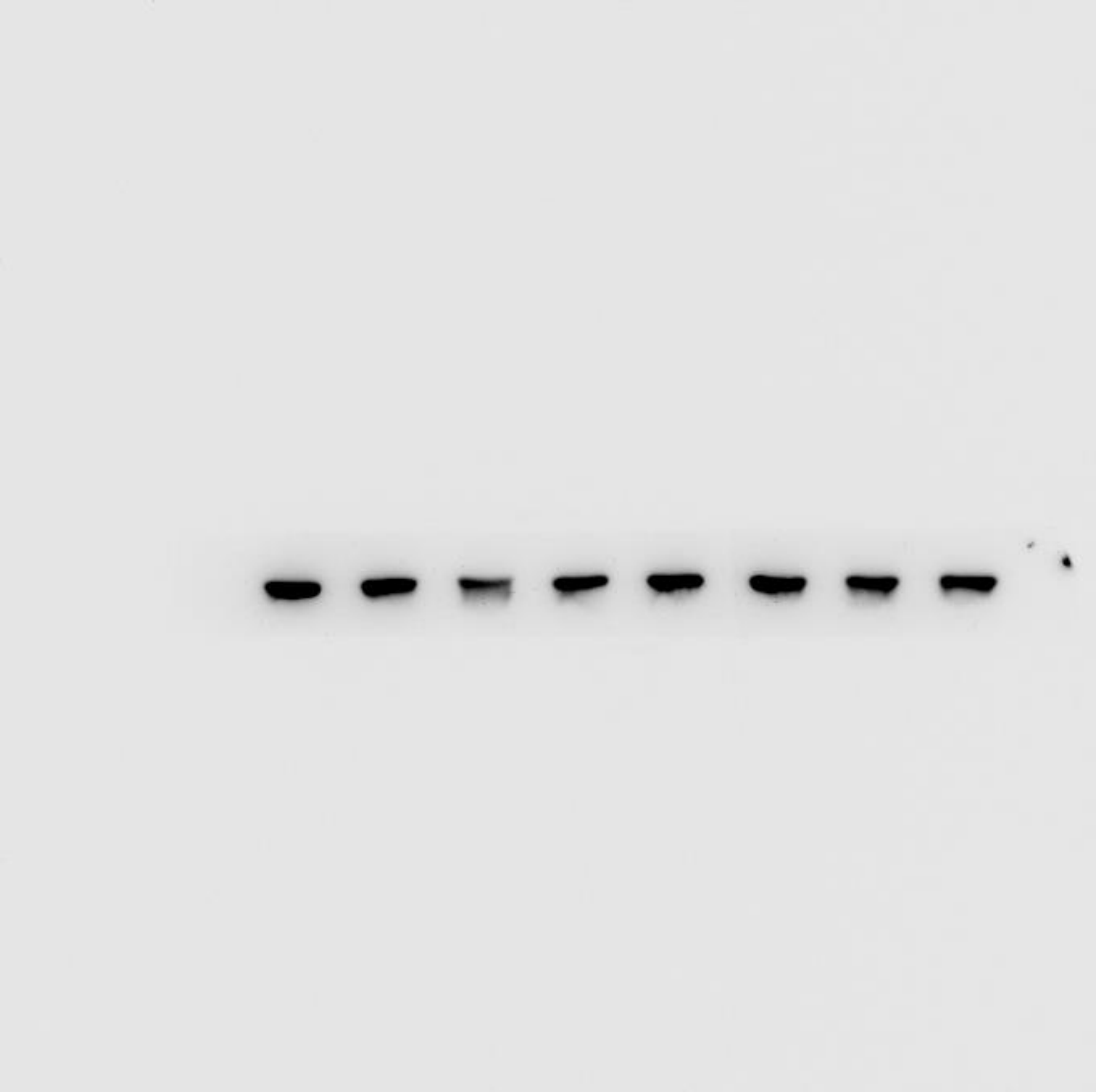

Figure_2A
CKAP2L

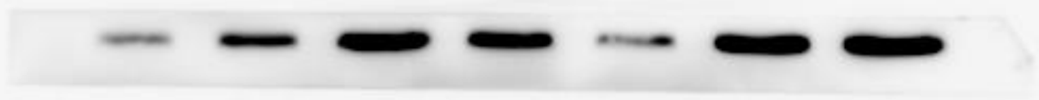

Figure_2A
GAPDH

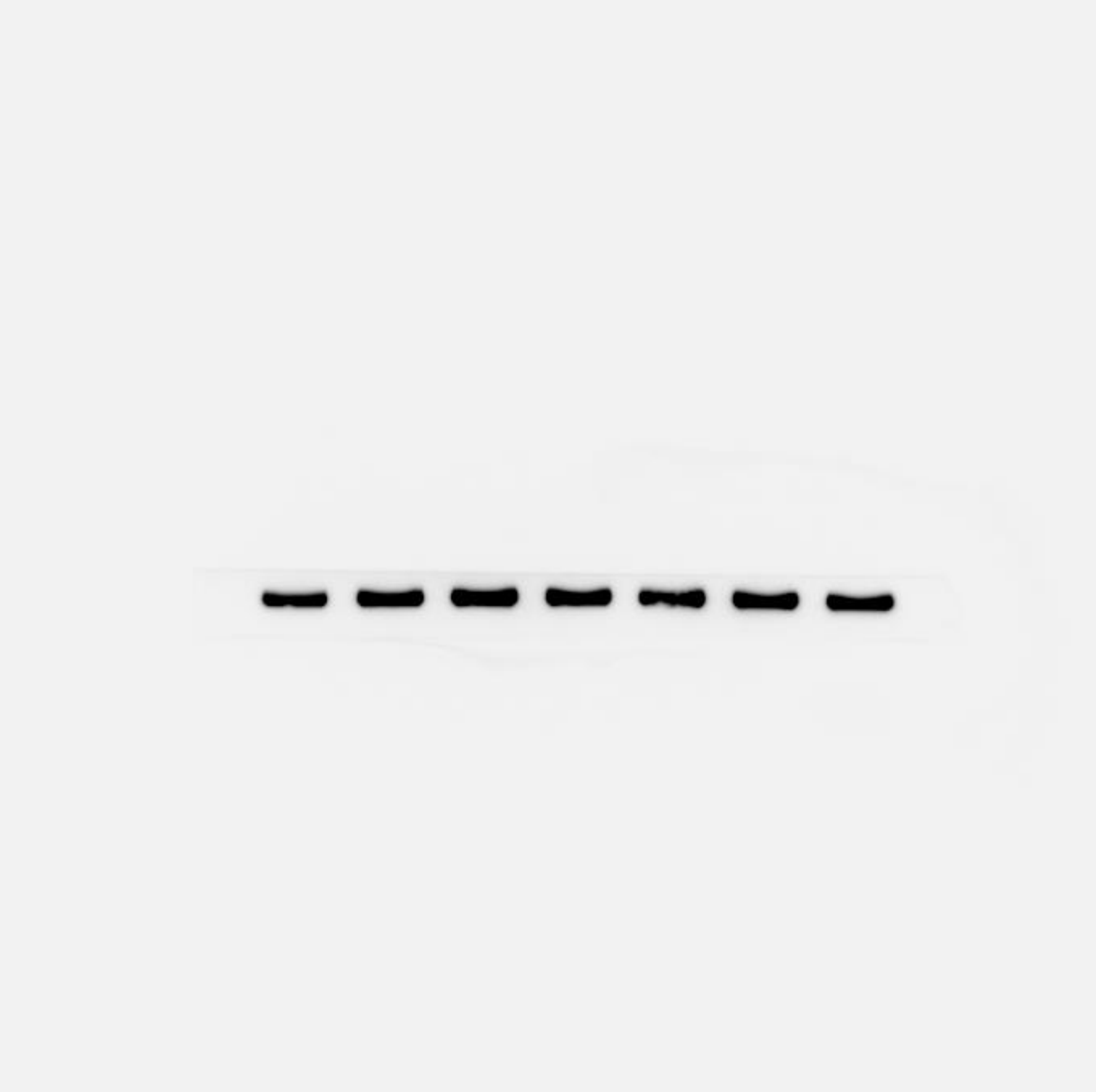

Figure_2B
CKAP2L
1-4 were used
5-8 were duplicates

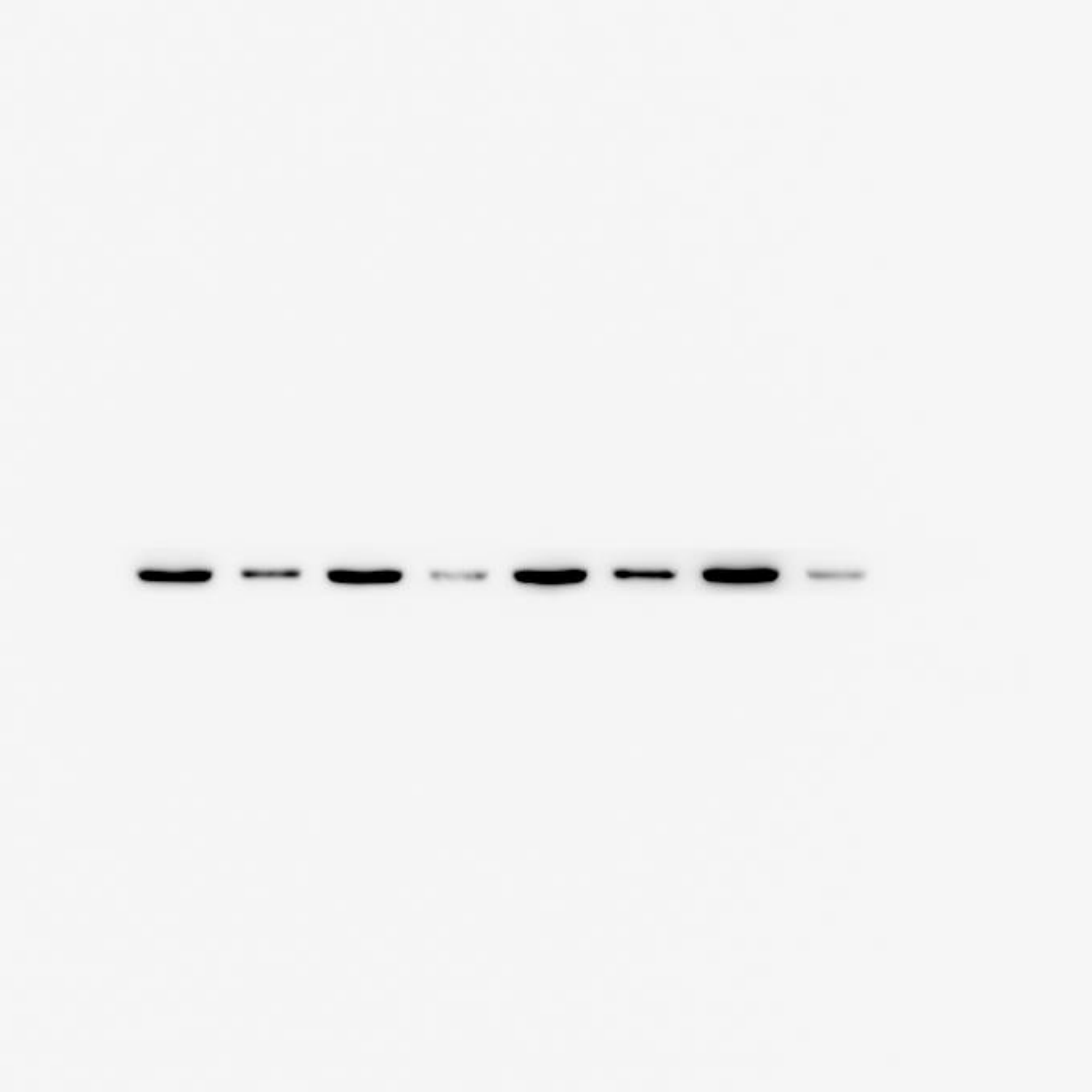

Figure_2B
GAPDH
1-4 were used
5-8 were duplicates

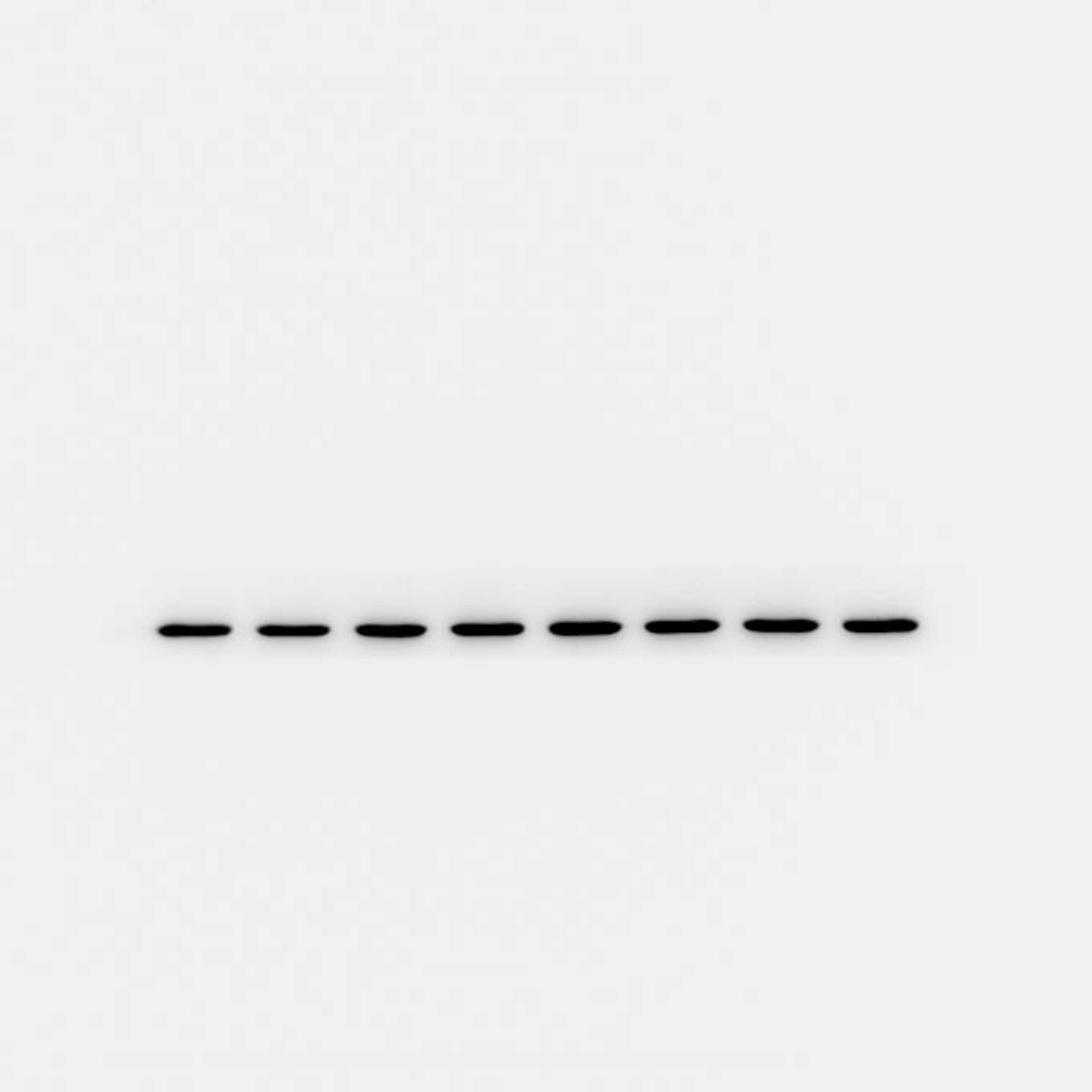

Figure_3E
CKAP2L
1-4 were used
5-8 were duplicates

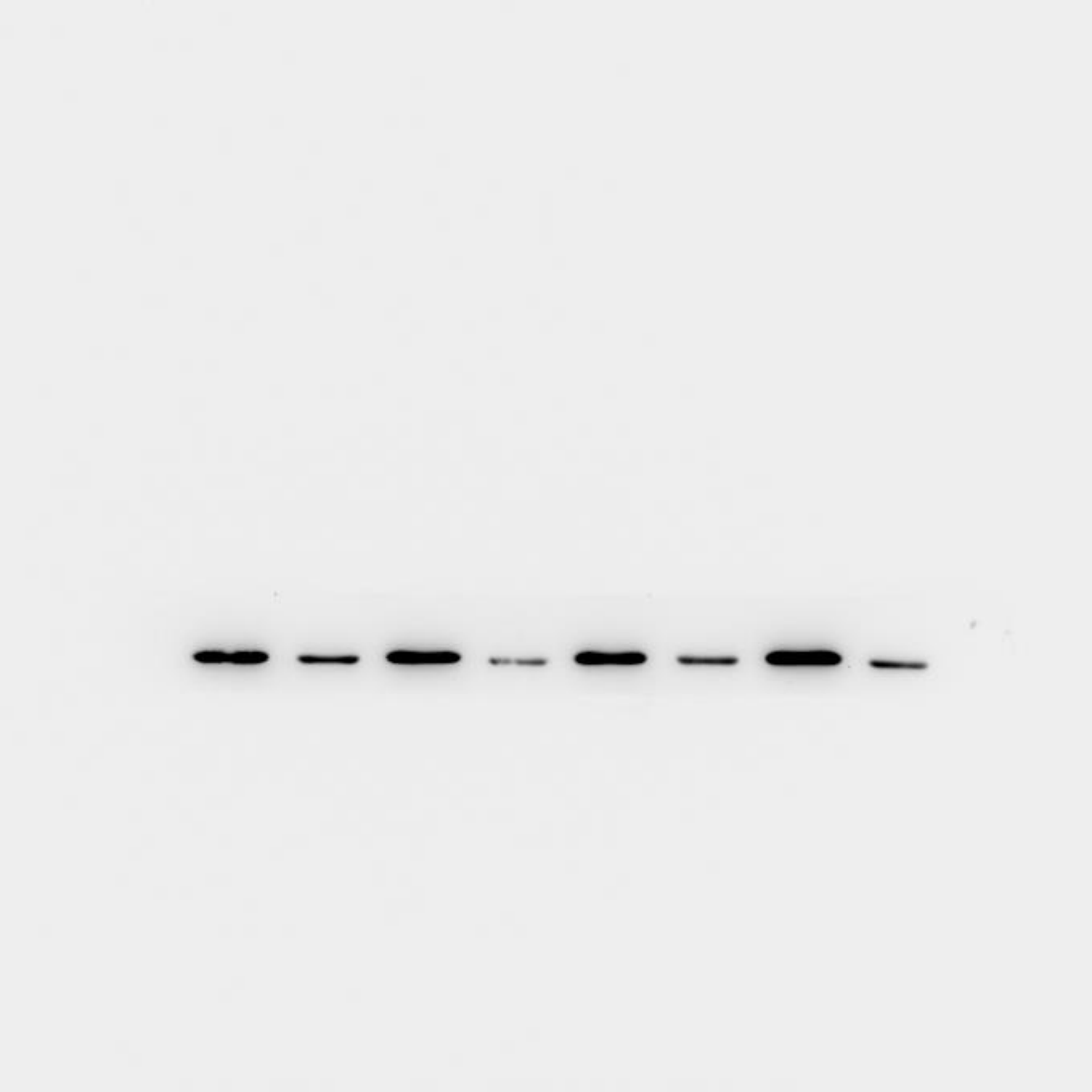

Figure_3E
GAPDH
1-4 were used
5-8 were duplicates

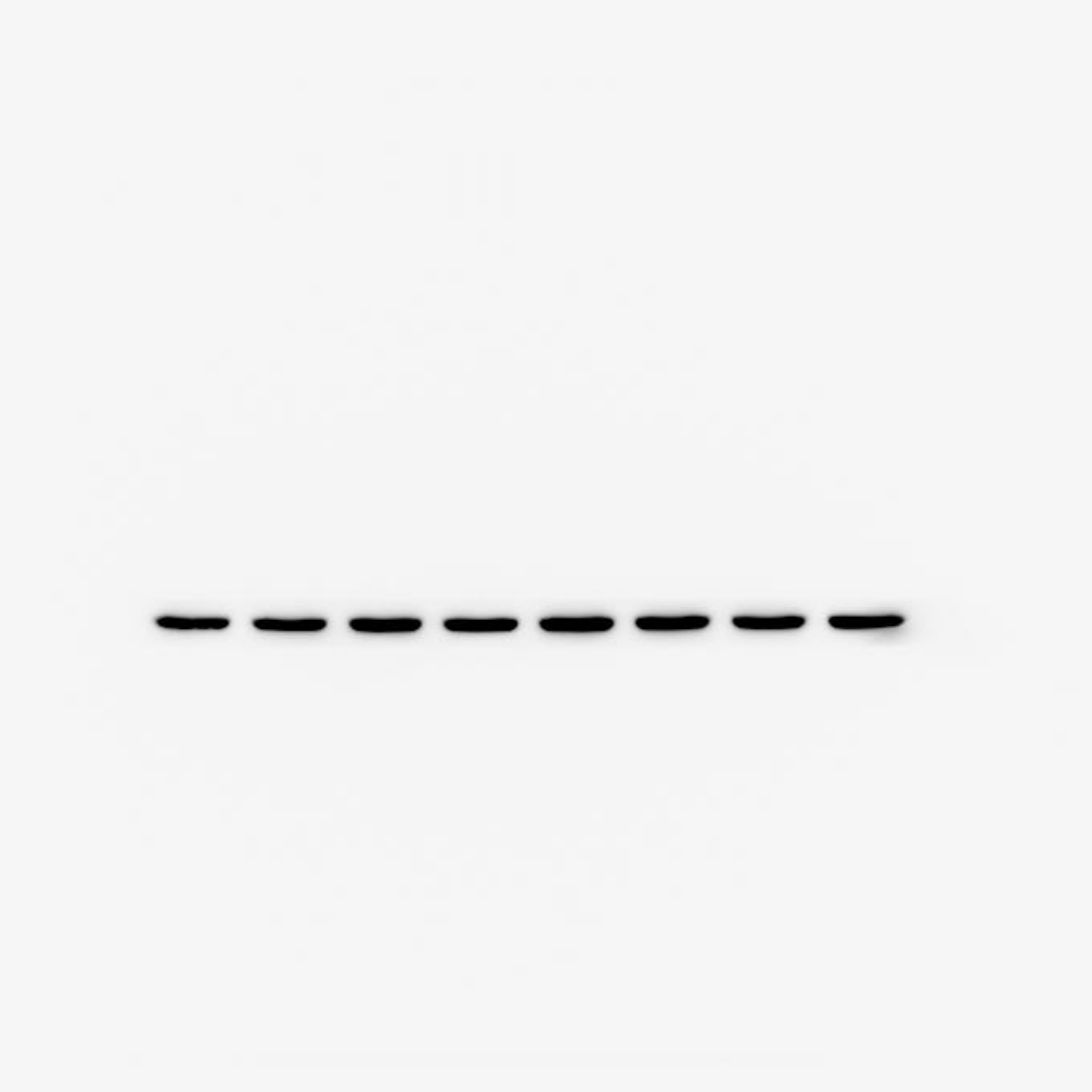

Figure_4G
BIRC5

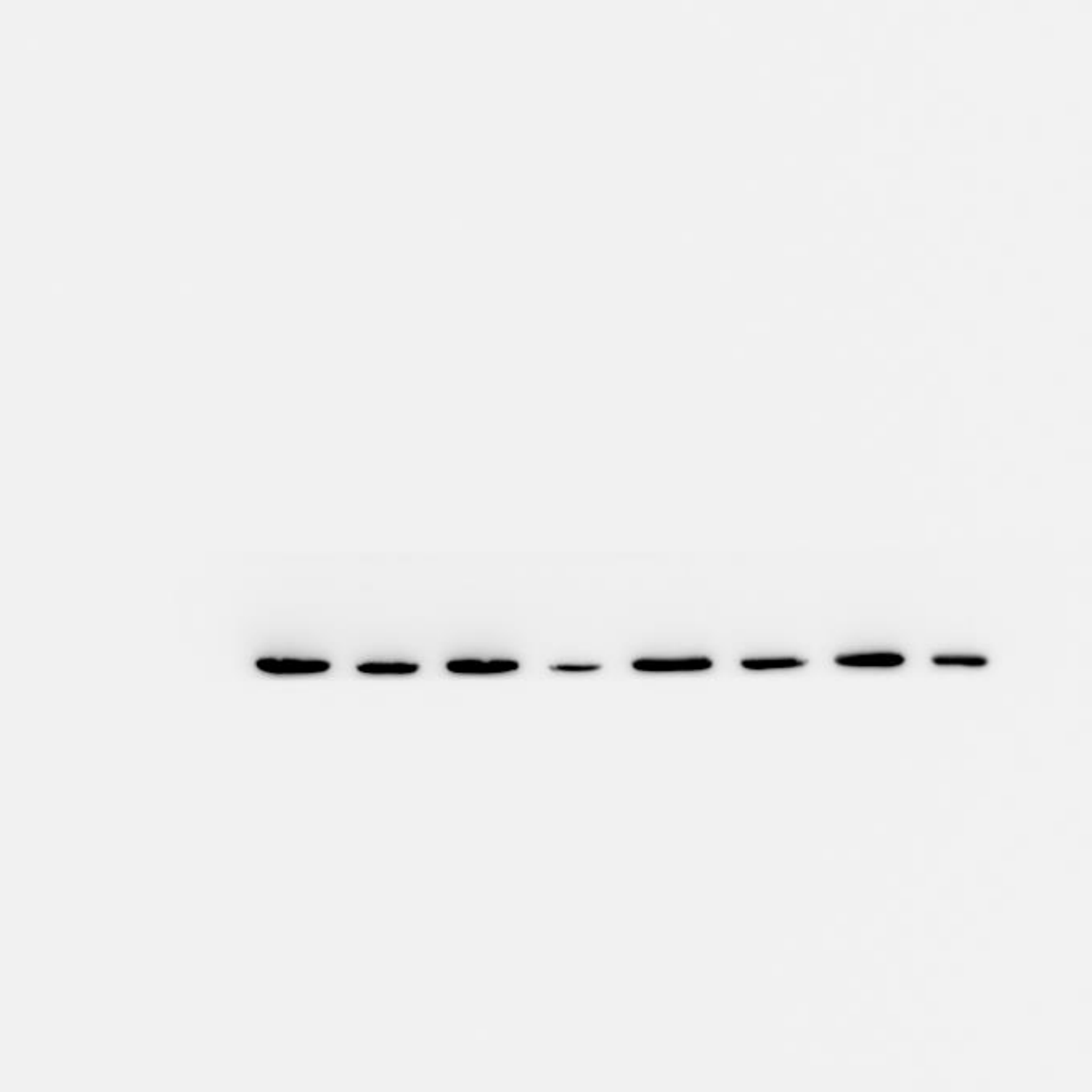

Figure_4G
MAD2L1

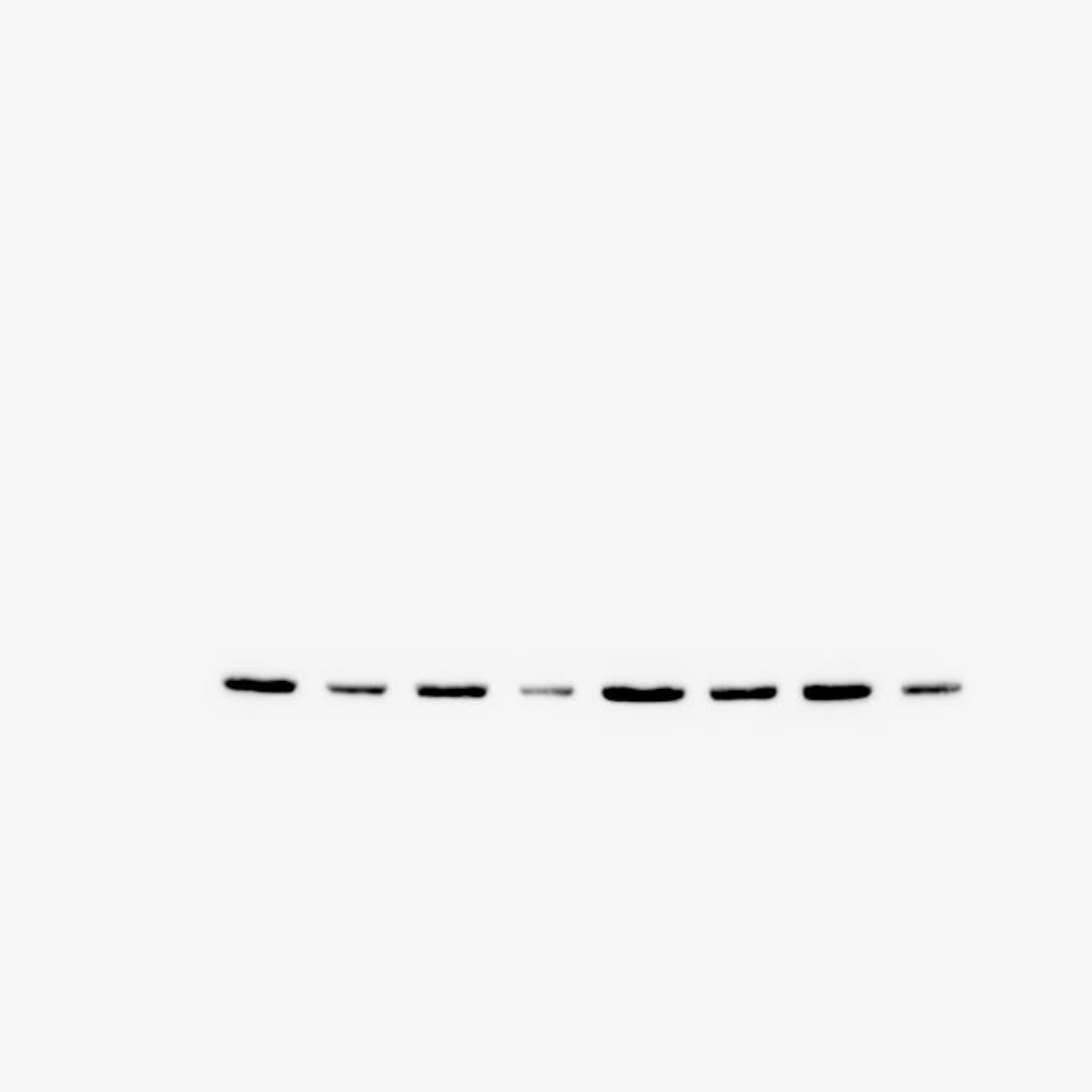

Figure_4G
CCND1

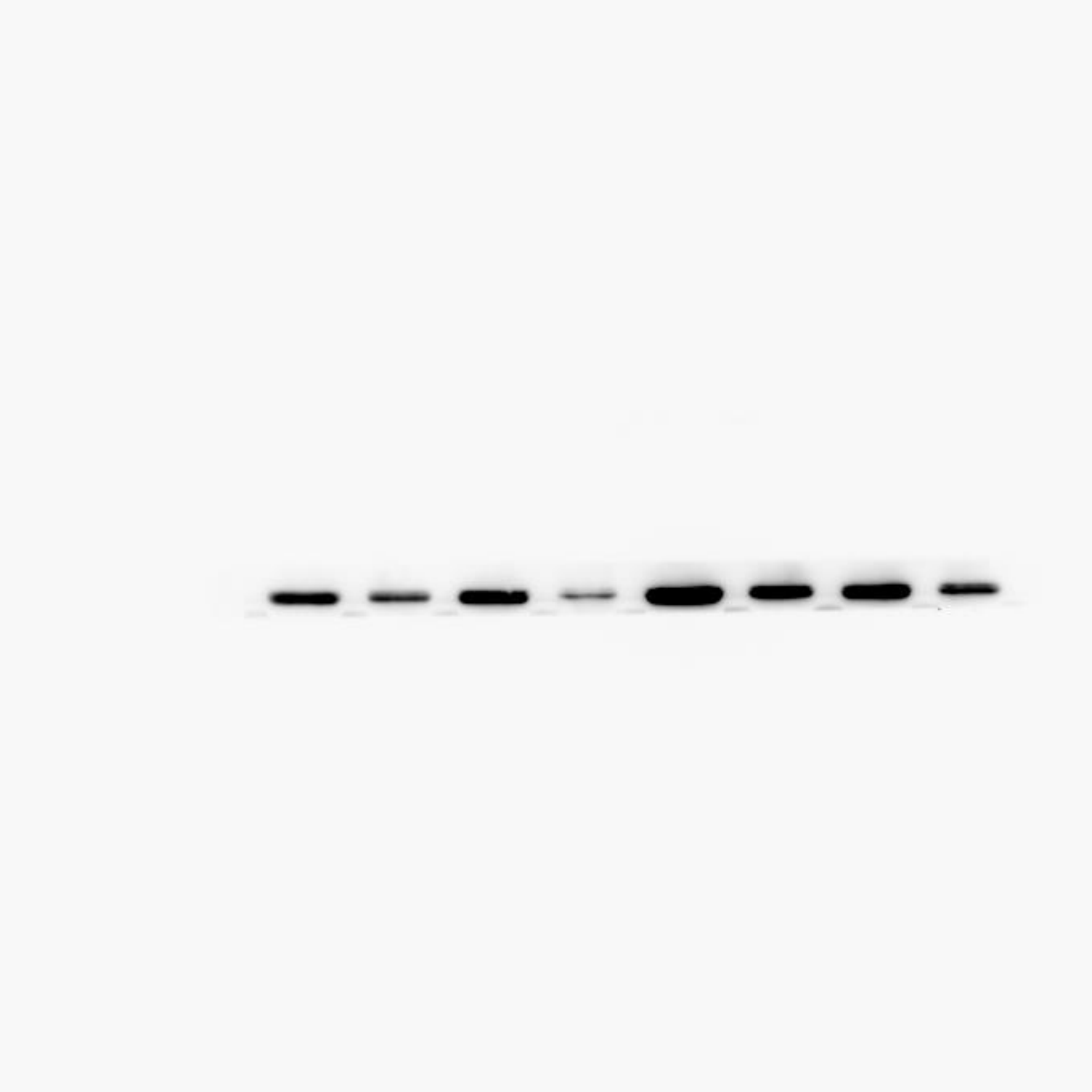

Figure_4G
AURKB

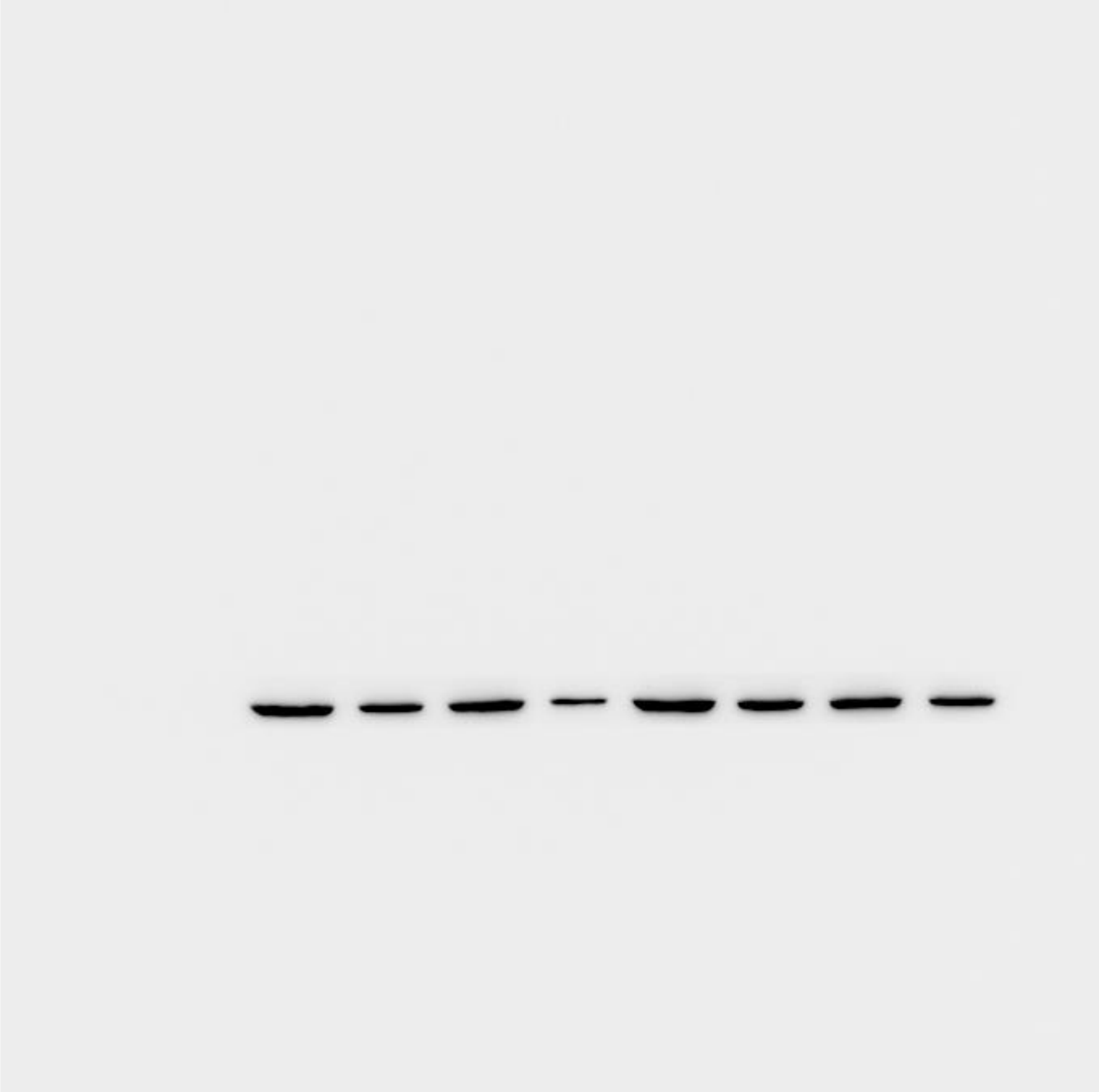

Figure_4G
PLK1

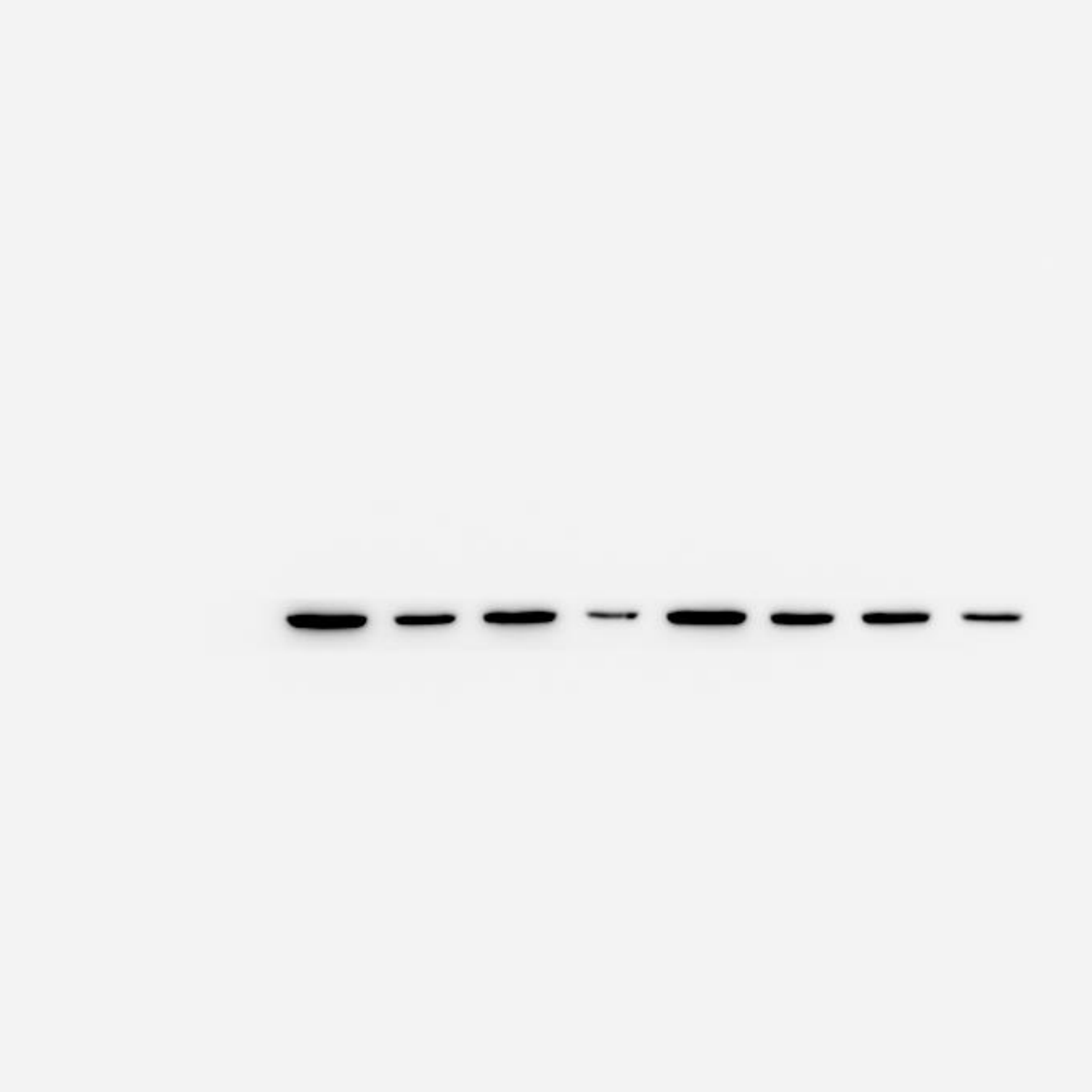

Figure_4G
KIF2C

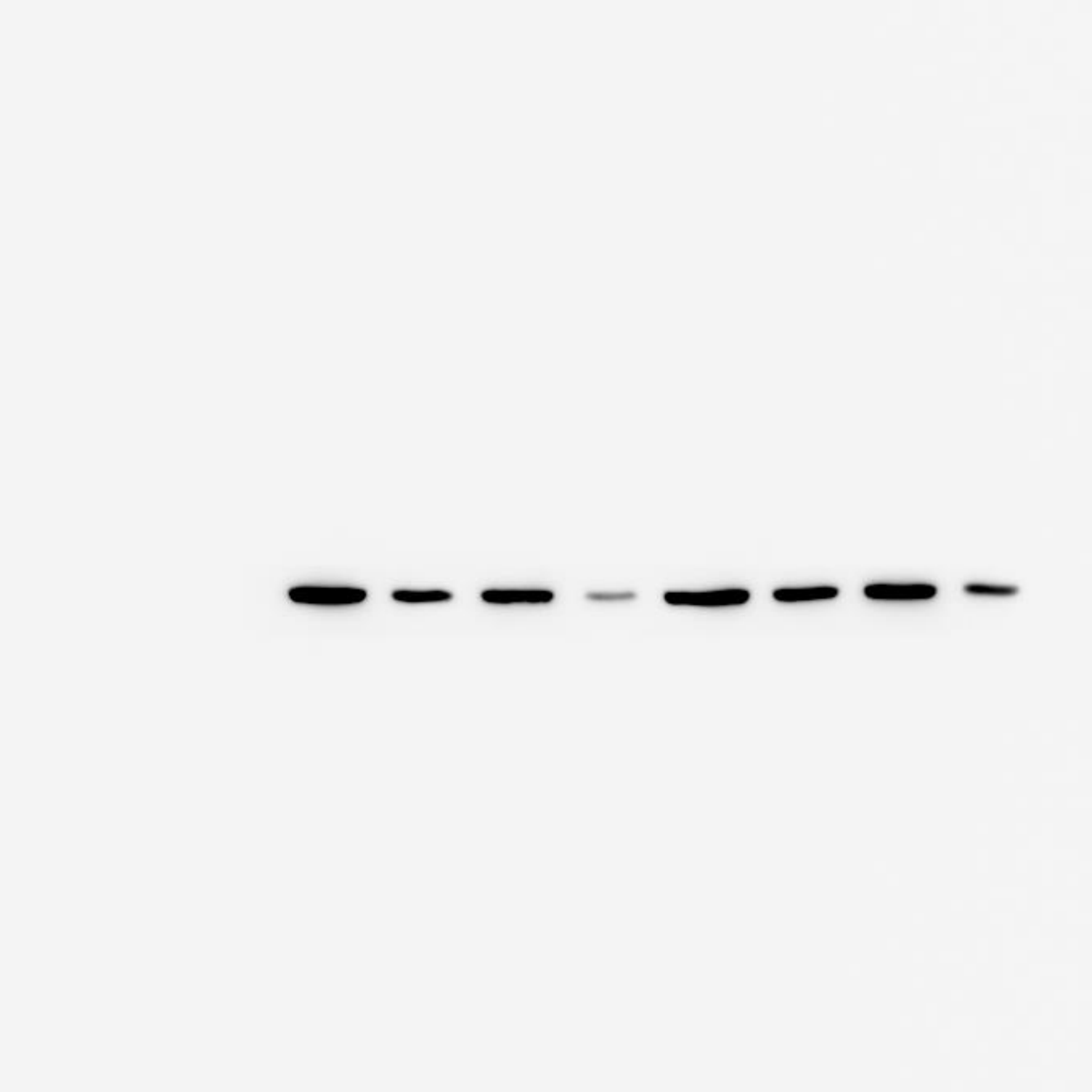

Figure_4G
SMC4

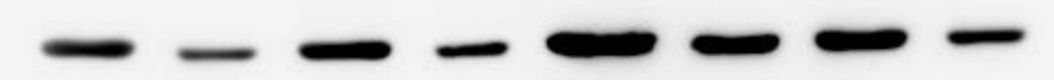

Figure_4G
GAPDH

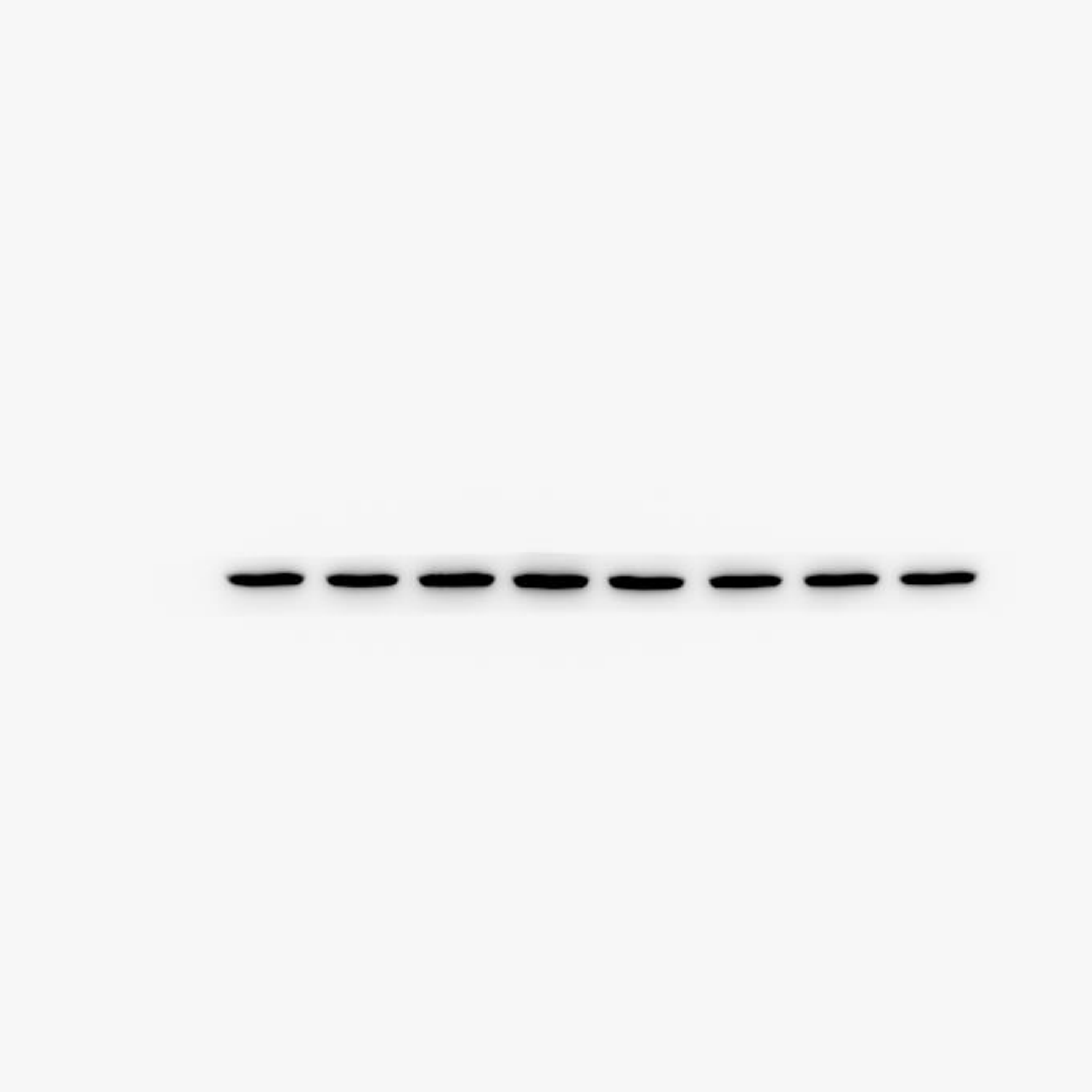

Figure_5B
CKAP2L_LNCaP-AI

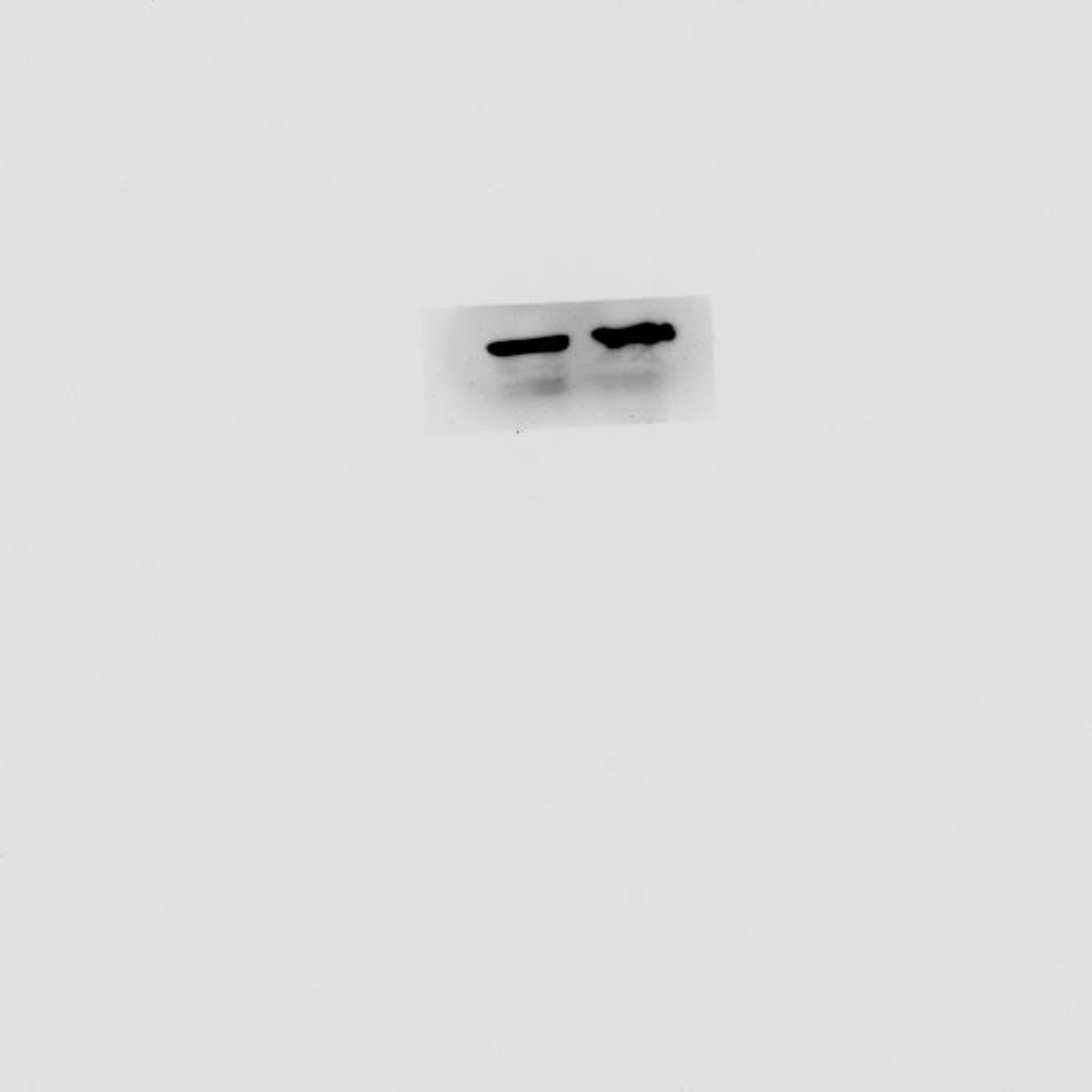

Figure_5B
CKAP2L_PC-3

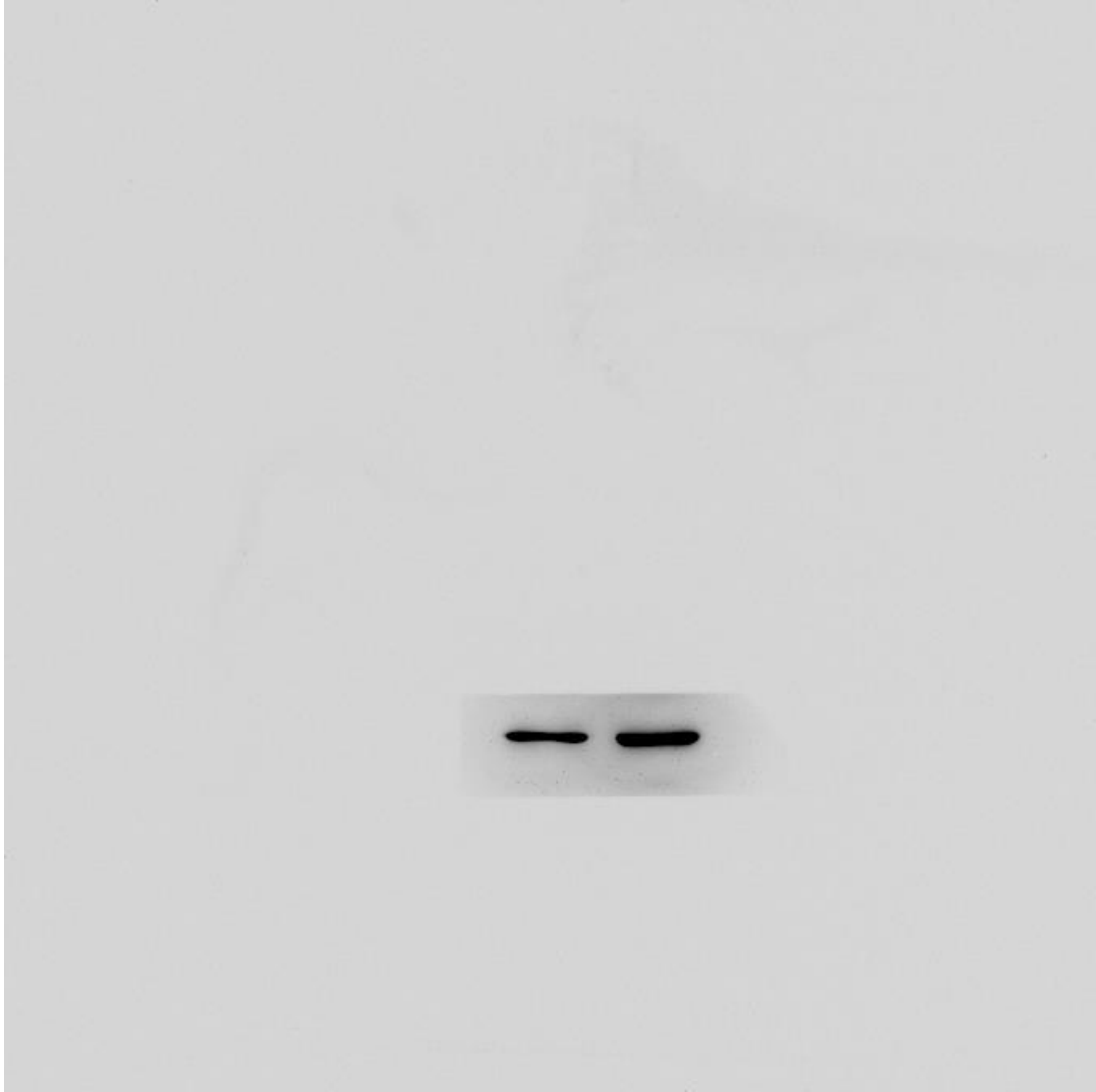

Figure_5B
GAPDH_LNCaP-AI

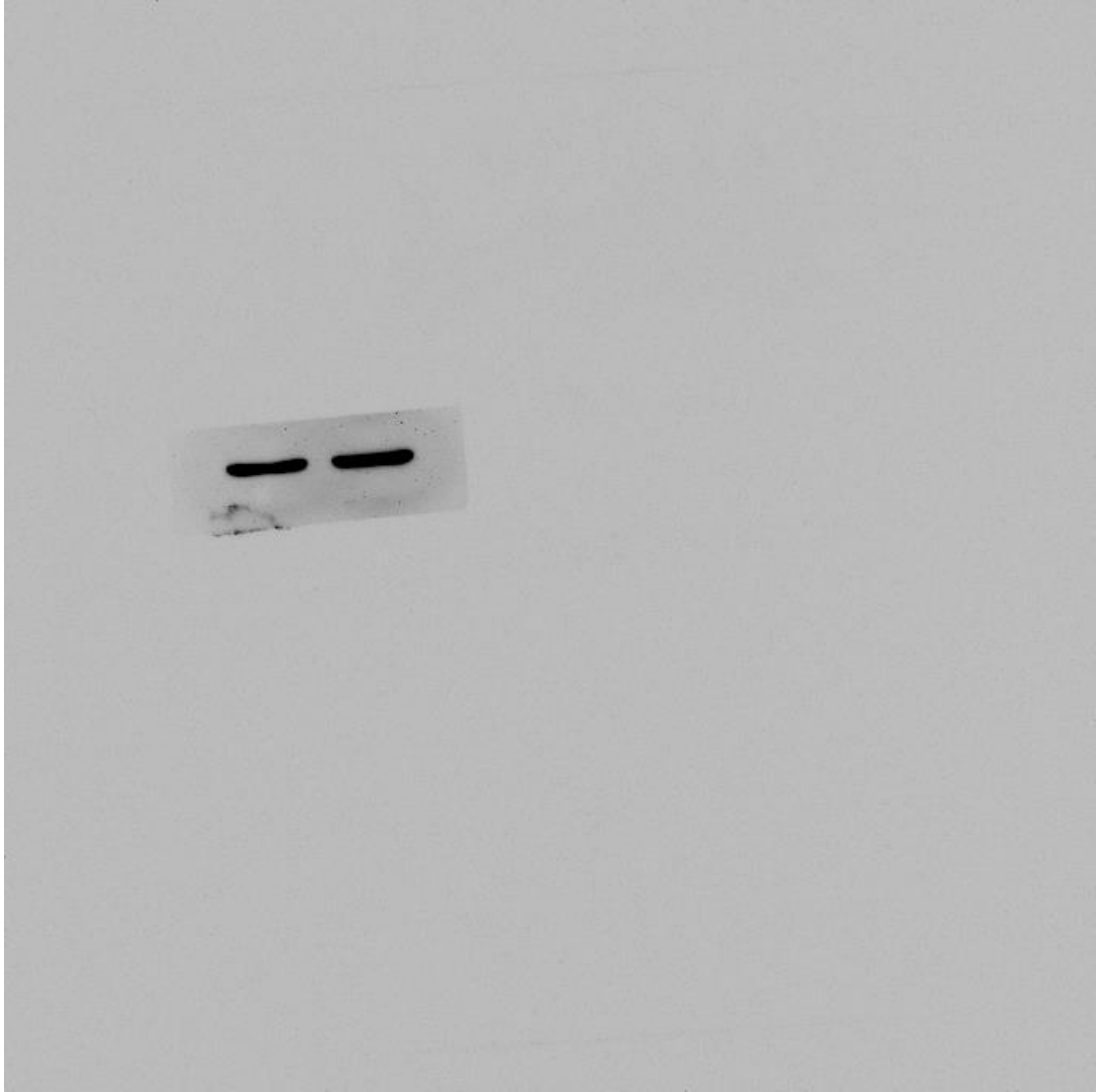

Figure_5B
GAPDH_PC-3

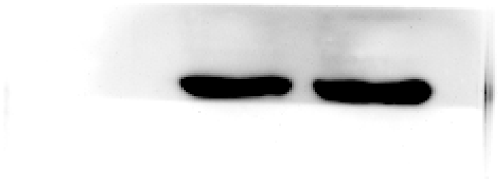

Supplement: Supplementary file 2 — Additional file 2: (PDF 235 kb) [file 12885_2022_9762_MOESM2_ESM.pdf]
